# Global Taxonomy and Phylogeny of Irpicaceae (Polyporales, Basidiomycota) With Descriptions of Seven New Species and Proposals of Two New Combinations

**DOI:** 10.3389/fmicb.2022.911978

**Published:** 2022-06-20

**Authors:** Yue Li, Shuang-Hui He, Che-Chih Chen, Karen K. Nakasone, Hai-Xia Ma

**Affiliations:** ^1^School of Ecology and Nature Conservation, Beijing Forestry University, Beijing, China; ^2^Department of Biology, National Museum of Natural Science, Taichung, Taiwan; ^3^Biodiversity Research Center, Academia Sinica, Taipei, Taiwan; ^4^Center for Forest Mycology Research, Northern Research Station, U.S. Forest Service, Madison, WI, United States; ^5^Institute of Tropical Bioscience and Biotechnology, Chinese Academy of Tropical Agricultural Sciences, Haikou, China

**Keywords:** corticioid fungi, East Asia, *Phanerochaete* s.l., phlebioid clade, white rot, wood-decaying fungi

## Abstract

The phylogenetic analyses of the family Irpicaceae were carried out based on a complete global sampling. The dataset that included concatenated ITS1-5.8S-ITS2 and nrLSU sequences of 67 taxa of Irpicaceae from around the world was subjected to the maximum likelihood analyses and Bayesian inference. In the phylogenetic tree, species from 14 genera were distributed in nine clades, among which five genera—*Irpex, Phanerochaetella, Byssomerulius, Cytidiella*, and *Meruliopsis*, received high support values. The genus *Efibula* was shown to be paraphyletic and four subclades could be recognized, while *Phanerochaete allantospora, Leptoporus mollis*, and several species from *Ceriporia* and *Candelabrochaete* formed a large clade with relatively strong support. Based on the molecular and morphological evidence, seven new corticioid species**—***Candelabrochaete guangdongensis, Efibula grandinosa, E. hainanensis, E. shenghuae, E. taiwanensis, Irpex alboflavescens*, and *Phanerochaetella sinensis*, were revealed from the materials mostly from East Asia. The monotypic genus *Flavodontia*, newly described from southwestern China, is regarded as a later synonym of *Irpex*, and the new combination *I. rosea* is proposed. In addition, *Phanerochaetella queletii* is proposed for a taxon first described from Italy and newly recorded from China; *Phanerochaete jose-ferreirae* from Portugal is determined to be a later synonym. Descriptions and illustrations of the new species and the newly combined taxa are presented, and morphological comparisons for the known species of *Efibula* and *Phanerochaetella* are provided.

## Introduction

The phlebioid clade of Polyporales includes lots of wood-decaying fungi, which were distributed in the three well-supported families: Phanerochaetaceae, Irpicaceae, and Meruliaceae (Floudas and Hibbett, 2015; Miettinen et al., 2016; Justo et al., 2017; Chen et al., 2021). Irpicaceae is a relatively new and small family with 13 corticioid and polyporoid genera accepted at present (Spirin, 2003): *Byssomerulius* Parmasto, *Ceriporia* Donk, *Crystallicutis* El-Gharabawy, Leal-Dutra and G.W. Griff, *Cytidiella* Pouzar, *Efibula* Sheng H. Wu, *Gloeoporus* Mont., *Irpex* Fr., *Leptoporus* Quél., *Meruliopsis* Bondartsev, *Phanerochaetella* C.C. Chen and Sheng H. Wu, *Raduliporus* Spirin and Zmitr., *Resiniporus* Zmitr., and *Trametopsis* Tomšovský. Species in the family usually have resupinate to effused-reflexed basidiomata, a monomitic hyphal system with simple-septate generative hyphae, colorless, thin-walled, and smooth basidiospores without reactions in cotton blue and Melzer's reagents, and lack cystidia. Ecologically, members of the Irpicaceae are widely distributed from temperate to tropic areas mostly on woody angiosperms and associated with a white rot decay, except for *Leptoporus* species which are associated with brown-rot decay (Chen et al., 2021).

*Irpex* was created in 1,825 by Fries and is the generic type of Irpicaceae which included 13 genera. Over time, species with hydnoid to irpicioid and even poroid hymenophores were placed in *Irpex*. By narrowing the morphological criteria for the genus, species were transferred to other genera and *Irpex* evolved into a morphologically well-defined genus with supporting phylogenetic evidence. Ryvarden (2020) evaluated the taxonomic status of 180 names described in *Irpex* and restricted the genus to two species, *I. lacteus* (Fr.) Fr. and *I. hydnoides* Y.W. Lim and H.S. Jung. Recently, Chen et al. (2021) showed by phylogenetic analyses of multiple genes that *Emmia* Zmitr., Spirin and Malysheva, *Flavodon* Ryvarden and *Hydnopolyporus* D.A. Reid and *Irpex* formed a strongly supported clade and placed the first three genera in synonymy under *Irpex*. They also showed that the genus *Efibula* was paraphyletic in the phylogenetic tree but lacked sufficient morphological evidence to support the recognition of separate genera (Chen et al., 2021). The polyphyletic genus *Ceriporia* was distributed in the three families of the phlebioid clade with the type species nested within Irpicaceae. Although lineages in Phanerochaetaceae and Meruliaceae have been resolved, the relationship between *Ceriporia* s.s. and species of *Candelabrochaete* s.l. requires further studies (Miettinen et al., 2016; Chen et al., 2020, 2021).

A large number of specimens belonging to Irpicaceae were collected from East Asia by the corresponding author in recent years. In order to further the knowledge of species diversity and taxonomy of Irpicaceae, we carried out complete morphological and molecular phylogenetic studies on our specimens with emphasis on corticioid taxa, and our results are presented below.

## Materials and Methods

### Specimen Collection

Field trips for specimen collection in many kinds of Nature Reserves and Forest Parks in China and other countries were carried out by the authors. *In situ* photographs of the fungi were taken with a Canon camera EOS 70D (Canon Corporation, Japan). Fresh specimens were dried with a portable drier (manufactured in Finland). Dried specimens were labeled and then stored in a refrigerator at minus 40 °C for two weeks to kill the insects and their eggs before they were ready for morphological and molecular studies.

### Morphological Studies

Voucher specimens are deposited at the herbaria of Beijing Forestry University, Beijing, China (BJFC), Centre for Forest Mycology Research, U.S. Forest Service, Madison, Wisconsin, USA (CFMR), and National Museum of Natural Science, Taichung, Taiwan, China (TNM). Herbarium code designations follow Index Herbarium^1^ Thin, freehand sections were made from dried basidiomata and mounted in 2% (w/v) aqueous potassium hydroxide (KOH) and 1% (w/v) aqueous phloxine. Amyloidity and dextrinoidity of basidiospores were checked in Melzer's reagent (IKI). Cyanophily of hyphal and basidiospore walls was observed in 1% (weight/volume) cotton blue in 60% (w/v) lactic acid (CB). Microscopic examinations were carried out with a Nikon Eclipse 80i microscope (Nikon Corporation, Japan) at magnifications up to 1,000 ×. Drawings were made with the aid of a drawing tube. The following abbreviations are used: IKI– = neither amyloid nor dextrinoid, CB– = acyanophilous, L = mean spore length, W = mean spore width, Q = L/W ratio, n (a/b) = number of spores (a) measured from number of specimens (b). Color codes and names follow Kornerup and Wanscher (1978).

### DNA Extraction and Sequencing

A CTAB plant genomic DNA extraction Kit DN14 (Aidlab Biotechnologies Co., Ltd, Beijing, China) was used to extract total genomic DNA from dried specimens and then amplified by the polymerase chain reaction (PCR), according to the manufacturer's instructions. The ITS1-5.8S-ITS2 region was amplified with the primer pair ITS5/ITS4 (White et al., 1990) using the following protocol: initial denaturation at 95 °C for 4 min, followed by 34 cycles at 94 °C for 40 s, 58 °C for 45 s and 72 °C for 1 min, and final extension at 72 °C for 10 min. The nrLSU D1-D2 region was amplified with the primer pair LR0R/LR7^2^ employing the following procedure: initial denaturation at 94 °C for 1 min, followed by 34 cycles at 94 °C for 30 s, 50 °C for 1 min and 72 °C for 1.5 min, and final extension at 72 °C for 10 min. DNA sequencing was performed at Beijing Genomics Institute, and the sequences were deposited in GenBank^3^ (Table 1). BioEdit v.7.0.5.3 (Hall, 1999) and Geneious Basic v.11.1.15 (Kearse et al., 2012) were used to review the chromatograms and for contig assembly.

**Table 1:** Species and sequences used in the phylogenetic analyses.

**Taxa**	**Voucher**	**Locality**	**ITS**	**nrLSU**	**Reference**
*Byssomerulius corium*	FP-102382	USA	KP135007	KP135230	Floudas and Hibbett, 2015
*Byssomerulius corium*	WEI 17-645	China	LC427006	LC427030	Chen et al., 2020
***Candelabrochaete guangdongensis***	He 5902*	**China**	**MZ422527**	**MZ422499**	**Present study**
*Candelabrochaete langloisii*	FP-110343-Sp	USA	KY948793	KY948886	Justo et al., 2017
*Candelabrochaete septocystidia*	AS-95	Sweden	EU118609	EU118609	Larsson, 2007
*Candelabrochaete septocystidia*	RLG-9759-Sp	USA	—	GQ470631	Wu et al., 2010
*Candelabrochaete septocystidia*	RMJ119sp	USA	KY948783	—	Justo et al., 2017
*Ceriporia arbuscula*	GC 1708-338	China	LC427008	LC427040	Chen et al., 2020
*Ceriporia mellita*	GC 1508-71	China	LC427022	LC427044	Chen et al., 2020
*Ceriporia mellita*	WEI 17-024	China	LC427024	LC427046	Chen et al., 2020
*Ceriporia purpurea*	GC 1703-81	China	LC427020	LC427037	Chen et al., 2020
*Ceriporia reticulata*	RLG-11354-Sp	USA	KP135041	KP135204	Floudas and Hibbett, 2015
*Ceriporia reticulata*	Wu 1707-171	China	LC427021	LC427038	Chen et al., 2020
*Ceriporia viridans*	GC 1708-211	China	LC427027	LC427049	Chen et al., 2020
*Ceriporia viridans*	Miettinen 11701	Netherlands	KX752600	KX752600	Miettinen et al., 2016
*Crystallicutis damiettensis*	UN63A	Egypt	KX428470	—	El-Gharabawy et al., 2016
*Crystallicutis rajchenbergii*	MR-4310	USA	KY948797	KY948888	Justo et al., 2017
*Crystallicutis serpens*	HHB-15692-Sp	USA	KP135031	KP135200	Floudas and Hibbett, 2015
*Crystallicutis* sp.	FP-101245-Sp	USA	KP135029	—	Floudas and Hibbett, 2015
*Cytidiella albida*	GB-1833	Spain	KY948748	KY948889	Justo et al., 2017
*Cytidiella albomarginata*	He 5575	China	MZ422526	MZ422497	Present study
*Cytidiella albomarginata*	WEI 18-474	China	MZ636948	MZ637110	Chen et al., 2021
*Cytidiella albomellea*	FP-101843-sp	USA	AY219369	—	De Koker et al., 2003
*Cytidiella albomellea*	He 3089	China	MZ422525	MZ422496	Present study
*Cytidiella nitidula*	He 5126	China	MZ422523	MZ422494	Present study
*Cytidiella nitidula*	He 5135	China	MZ422524	MZ422495	Present study
*Cytidiella nitidula*	Nystroem 020830	Sweden	EU118655	EU118655	Larsson, 2007
*Cytidiella nitidula*	T-407	Canada	KY948747	—	Justo et al., 2017
*Cytidiella* sp.	He 6198	China	—	MZ422498	Present study
*Cytidiella* sp.	Wu 0010-171	China	MZ636951	MZ637114	Chen et al., 2021
*Cytidiella* sp.	Wu 1409-168	China	MZ636952	MZ637115	Chen et al., 2021
*Efibula americana*	FP-102165	USA	KP135016	KP135256	Floudas and Hibbett, 2015
*Efibula americana*	HHB-10209-Sp	USA	KP135014	—	Floudas and Hibbett, 2015
*Efibula clarkii*	FD-228	USA	KP135019	—	Floudas and Hibbett, 2015
*Efibula gracilis*	FD-455	USA	KP135027	—	Floudas and Hibbett, 2015
*Efibula gracilis*	FP-102052	USA	KP135028	—	Floudas and Hibbett, 2015
***Efibula grandinosa***	He 6312s*	**China**	**MZ422509**	**MZ422480**	**Present study**
***Efibula hainanensis***	He 6004*	**China**	**MW580949**	**MW580939**	**Present study**
***Efibula hainanensis***	**Chen 1284**	**China**	**ON117184**	**—**	**Present study**
*Efibula intertexta*	Wu 1707-96	China	MZ636954	MZ637118	Chen et al., 2021
*Efibula matsuensis*	Chen 1510	China	MZ636955	—	Chen et al., 2021
***Efibula shenghuae***	He 3384*	**China**	**MZ422508**	**MZ422479**	**Present study**
*Efibula* sp.	FCUG 305	Sweden	MZ636959	GQ470669	Wu et al., 2010; Chen et al., 2021
*Efibula* sp.	Wu 1707-79	China	MZ636961	MZ637123	Chen et al., 2021
*Efibula subglobispora*	Chen 1716	China	MZ636962	MZ637124	Chen et al., 2021
*Efibula subglobispora*	He 3983	China	MW580944	MW580934	Present study
*Efibula subglobispora*	He 7032	China	MZ422506	MZ422477	Present study
***Efibula taiwanensis***	He 4582a*	**China**	**MZ422507**	**MZ422478**	**Present study**
*Efibula tropica*	Chen 3596	China	MZ636966	MZ637128	Chen et al., 2021
*Efibula tropica*	He 6008	China	MW580947	MW580937	Present study
*Efibula tropica*	WEI 18-149	China	MZ636967	MZ637129	Chen et al., 2021
*Efibula tuberculata*	OM-6707	Finland	KP135017	—	Floudas and Hibbett, 2015
*Efibula tuberculata*	OM-11754	Finland	KP135018	—	Floudas and Hibbett, 2015
*Efibula turgida*	He 3145	China	MW580945	MW580935	Present study
*Efibula turgida*	He 6711	China	MW580946	MW580936	Present study
*Efibula turgida*	Wu 0910-99	China	MZ636973	MZ637135	Chen et al., 2021
*Efibula yunnanensis*	CLZhao 11641	China	MT611529	—	Ma et al., 2020
*Efibula yunnanensis*	He 4653	China	MW580948	MW580938	Present study
*Efibula yunnanensis*	He 6970	China	MZ422505	MZ422476	Present study
*Hapalopilus ochraceolateritius*	Miettinen 16992	USA	KY948741	KY948891	Justo et al., 2017
***Irpex alboflavescens***	**FP-160003**	**USA**	**KP135022**	**—**	**Floudas and Hibbett**, **2015**
***Irpex alboflavescens***	He 3933*	**China**	**MZ422503**	**MZ422474**	**Present study**
***Irpex alboflavescens***	**He 4719**	**China**	**MZ422501**	**MZ422472**	**Present study**
***Irpex alboflavescens***	**He 5783**	**Sri Lanka**	**MZ422500**	**MZ422471**	**Present study**
***Irpex alboflavescens***	**He 5835**	**Sri Lanka**	**MZ422504**	**MZ422475**	**Present study**
***Irpex alboflavescens***	**He 6355**	**Malaysia**	**MZ422502**	**MZ422473**	**Present study**
***Irpex alboflavescens***	**Wu 910807-35**	**China**	**MZ636994**	**GQ470627**	**Wu et al.**, **2010****; Chen et al.**, **2021**
*Irpex flavus*	LE295997	Tanzania	KF856505	KF856510	Zmitrovich and Malysheva, 2014
*Irpex flavus*	WHC 1381	China	LC427029	LC427052	Chen et al., 2020
*Irpex hydnoides*	KUC20121109-01	South Korea	KJ668510	KJ668362	Jang et al., 2016
*Irpex jinshaensis*	Dai 22402	China	MZ787973	MZ787965	Tian et al., 2022
*Irpex laceratus*	Dai 13638A	China	KX494576	KX494580	Yuan et al., 2017
*Irpex lacteus*	FD-9	USA	KP135026	KP135224	Floudas and Hibbett, 2015
*Irpex lacteus*	FD-93	USA	KP135025	**—**	Floudas and Hibbett, 2015
*Irpex latemarginatus*	FP-55521T	USA	KP135024	KP135202	Floudas and Hibbett, 2015
*Irpex latemarginatus*	Piatek 1997	Poland	KX752592	KX752592	Miettinen et al., 2016
*Irpex lenis*	Wu 1608-22	China	MZ636992	MZ637153	Chen et al., 2021
***Irpex rosea***	**He 6277**	**China**	**MW580943**	**MW580933**	**Present study**
***Irpex rosea***	CLZhao 18491*	**China**	**MW377575**	**MW377578**	**Wang and Zhao**, **2022**
***Irpex rosea***	**CLZhao 18489**	**China**	**MW377574**	**MW377577**	**Wang and Zhao**, **2022**
*Irpex rosettiformis*	LR40855	USA	JN649347	JN649347	Sjökvist et al., 2012
*Irpex rosettiformis*	Meijer3729	Brazil	JN649346	JN649346	Sjökvist et al., 2012
*Irpex subulatus*	BPI 893213	USA	NR_154000	NG_060421	Simmons et al., 2016
*Irpex subulatus*	CLZhao 3341	China	MH114652	—	Unpublished
*Irpex subulatus*	Cui 7275	China	KY131836	KY131895	Wu et al., 2017
*Irpex subulatus*	Dai 5929	China	KY131837	KY131896	Wu et al., 2017
*Irpex subulatus*	He 3468	China	MW580942	MW580932	Present study
*Leptoporus mollis*	RLG7163	USA	KY948794	**—**	Justo et al., 2017
*Leptoporus mollis*	TJV-93-174T	USA	KY948795	EU402510	Lindner and Banik, 2008; Justo et al., 2017
*Meruliopsis crassitunicata*	CHWC 1506-46	China	LC427010	LC427034	Chen et al., 2020
*Meruliopsis leptocystidiata*	Wu 1708-43	China	LC427013	LC427033	Chen et al., 2020
*Meruliopsis parvispora*	Wu 1209-58	China	LC427017	LC427039	Chen et al., 2020
*Meruliopsis taxicola*	GC 1704-60	China	LC427028	LC427050	Chen et al., 2020
*Phanerochaete allantospora*	KKN-111-Sp	USA	KP135038	KP135238	Floudas and Hibbett, 2015
*Phanerochaete allantospora*	RLG-10478	USA	KP135039	**—**	Floudas and Hibbett, 2015
*Phanerochaetella angustocystidiata*	He 4789	China	MZ422513	MZ422484	Present study
*Phanerochaetella angustocystidiata*	He 3167	China	MZ422514	MZ422485	Present study
*Phanerochaetella angustocystidiata*	He 2965	China	MZ422515	MZ422486	Present study
*Phanerochaetella angustocystidiata*	CLZhao 8393	China	MK404428	**—**	Unpublished
*Phanerochaetella angustocystidiata*	KUC20121102-15	South Korea	KJ668492	KJ668346	Unpublished
*Phanerochaetella angustocystidiata*	Wu 9606-39	China	**—**	GQ470638	Wu et al., 2010
*Phanerochaetella exilis*	HHB-6988-Sp	USA	KP135001	KP135236	Floudas and Hibbett, 2015
*Phanerochaetella formosana*	Chen 479	China	**—**	GQ470650	Wu et al., 2010
*Phanerochaetella formosana*	He 3391	China	MZ422520	MZ422491	Present study
*Phanerochaetella formosana*	He 3962	China	MZ422522	MZ422493	Present study
*Phanerochaetella formosana*	He 4411	China	MZ422521	MZ422492	Present study
*Phanerochaetella leptoderma*	103526	India	**—**	KP715577	Unpublished
*Phanerochaetella leptoderma*	Chen 1362	China	**—**	GQ470646	Wu et al., 2010
*Phanerochaetella leptoderma*	He 4770	China	MZ422516	MZ422487	Present study
***Phanerochaetella queletii***	**He 3050**	**China**	**MZ422512**	**MZ422483**	**Present study**
***Phanerochaetella queletii***	**He 3284**	**China**	**MZ422510**	**MZ422481**	**Present study**
***Phanerochaetella queletii***	**He 20120917-10**	**China**	**MZ422511**	**MZ422482**	**Present study**
***Phanerochaetella queletii***	**HHB-11463**	**USA**	**KP134994**	**KP135235**	**Floudas and Hibbett**, **2015**
***Phanerochaetella sinensis***	**He 3509**	**China**	**MZ422517**	**MZ422488**	**Present study**
***Phanerochaetella sinensis***	He 4229*	**China**	**MZ422518**	**MZ422489**	**Present study**
***Phanerochaetella sinensis***	**He 5071**	**China**	**MZ422519**	**MZ422490**	**Present study**
*Phanerochaetella* sp.	FP-102936	USA	KP135000	**—**	Floudas and Hibbett, 2015
*Phanerochaetella* sp.	HHB-18104	New Zealand	KP135003	KP135254	Floudas and Hibbett, 2015
*Phanerochaetella xerophila*	HHB-8509-Sp	USA	KP134996	KP135259	Floudas and Hibbett, 2015
*Phanerochaetella xerophila*	KKN-172-Sp	USA	KP134997	**—**	Floudas and Hibbett, 2015
*Raduliporus aneirina*	HHB-15629-Sp	USA	KP135023	KP135207	Floudas and Hibbett, 2015
*Resiniporus resinascens*	BRNM 710169	Czech Republic	FJ496675	FJ496698	Tomšovský et al., 2010
*Trametopsis aborigena*	Robledo 1236	Argentina	KY655336	KY655338	Lopes et al., 2017
*Trametopsis cervina*	TJV-93-216T	USA	JN165020	JN164796	Justo and Hibbett, 2011
**Outgroup**					
*Gloeoporus dichrous*	FP-151129	USA	KP135058	KP135213	Floudas and Hibbett, 2015
*Gloeoporus pannocinctus*	L-15726-Sp	USA	KP135060	KP135214	Floudas and Hibbett, 2015

New species and new combinations are set in bold with type specimens indicated with an asterisk *.

### Phylogenetic Analyses

The molecular phylogeny was inferred from a concatenated dataset of ITS-nrLSU sequences of species in the Irpicaceae. *Gloeoporus dichrous* (Fr.) Bres. and *G. pannocinctus* (Romell) J. Erikss. were selected as the outgroup (Floudas and Hibbett, 2015; Chen et al., 2021). The ITS and nrLSU sequences were aligned separately using MAFFT v.7^4^ (Katoh et al., 2017) with the G-INS-I iterative refinement algorithm and optimized manually in BioEdit v.7.0.5.3. The separate alignments were then concatenated using Mesquite v.3.5.1 (Maddison and Maddison, 2018). The datasets were deposited in TreeBase^5^ (submission ID: 29610).

Maximum likelihood (ML) analyses and Bayesian inference (BI) were carried out by using RAxML v.8.2.10 (Stamatakis, 2014) and MrBayes 3.2.6 (Ronquist et al., 2012), respectively. In ML analysis, statistical support values were obtained using rapid bootstrapping with 1000 replicates, with default settings used for other parameters. For BI, the best-fit substitution model was estimated with jModeltest v.2.17 (Darriba et al., 2012). Four Markov chains were run for 8,000,000 generations until the split deviation frequency value was lower than 0.01. Trees were sampled every 100th generation. The first quarter of the trees, which represented the burn-in phase of the analyses, were discarded, and the remaining trees were used to calculate posterior probabilities (BPP) in the majority rule consensus tree.

## Results

### Phylogenetic Analyses

**Figure 1:**
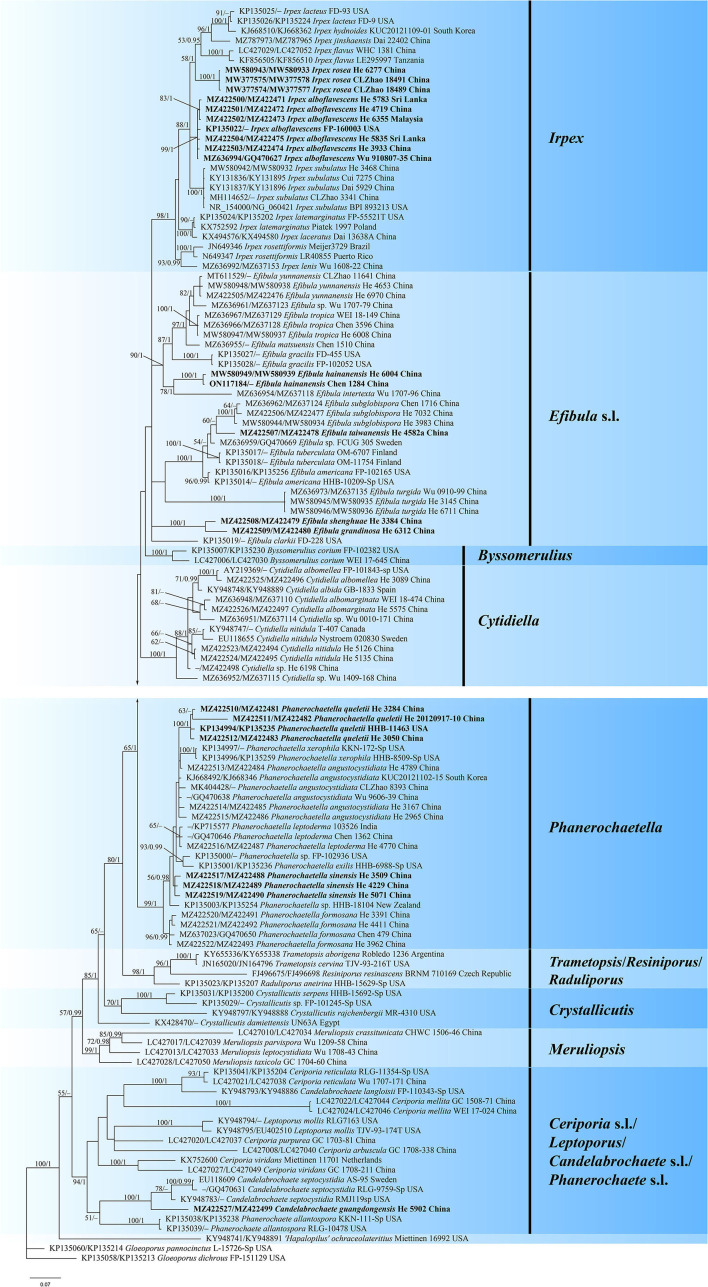
Phylogram of Irpicaceae inferred from Bayesian inference using the concatenated ITS-nrLSU sequences dataset. Branches are labeled with likelihood bootstrap values (≥50%, before the slash) and Bayesian posterior probabilities (≥0.95, after the slash). New species and new combinations are set in bold.

The concatenated ITS-nrLSU dataset contained 120 ITS and 101 nrLSU sequences from 126 samples representing 69 taxa of Irpicaceae (Table 1). The concatenated dataset had an aligned length of 2220 characters. jModelTest suggested that GTR+I+G was the best-fit model of nucleotide evolution for the concatenated ITS-nrLSU. The average standard deviation of split frequencies of BI was 0.007321 at the end of the run. ML analyses resulted in almost identical tree topology compared to the BI analysis. Only the BI tree is provided in Figure 1 with the likelihood bootstrap values (≥50%, before the slash) and Bayesian posterior probabilities (≥0.95, behind the slash) labeled along the branches.

The topology of the tree is similar to those in previous studies (Justo et al., 2017; Chen et al., 2021). For the in-groups, species from 14 genera were distributed in nine clades: *Irpex, Efibula, Phanerochaetella, Byssomerulius, Cytidiella, Raduliporus*/*Resiniporus*/*Trametopsis, Crystallicutis, Meruliopsis, Ceriporia* s.l./*Leptoporus/Candelabrochaete* s.l./ *Phanerochaete* s.l. While *Irpex, Phanerochaetella, Byssomerulius, Cytidiella*, and *Meruliopsis* received high support values, and the genus *Efibula* was shown to be polyphyletic. *Raduliporus, Resiniporus*, and *Trametopsis* formed a strongly supported clade. The monophyly of *Crystallicutis* was not well-supported in our tree. *Phanerochaete allantospora, Leptoporus mollis*, and several species from *Ceriporia* and *Candelabrochaete* formed a large clade with relatively strong support. Seven new species**—***Candelabrochaete guangdongensis, Efibula grandinosa, E. hainanensis, E. shenghuae, E. taiwanensis, Irpex alboflavescens*, and *P. sinensis*, formed distinct lineages in the tree. For *Irpex rosea*, our sample (He 6277) and the type materials of *Flavodontia rosea* (Zhao 18489 and Zhao 18491) formed a distinct lineage in the *Irpex* clade, while for *Phanerochaetella queletii*, samples from China and USA grouped together with strong support values.

### Taxonomy

#### *Candelabrochaete guangdongensis* Y. Li and S.H. He, sp. nov.

MycoBank: MB843531

Type—China, Guangdong Province, Shixing County, Chebaling Nature Reserve, on fallen angiosperm trunk, June 14, 2019, He 5902 (BJFC 030777, holotype).

Etymology—Refers to the type locality in Guangdong Province, southern China.

Fruiting body—Basidiomata annual, resupinate, widely effused, closely adnate, inseparable from substrate, ceraceous, first as small patches, later confluent up to 9-cm long, 3-cm wide, up to 250-μm thick in section. Hymenophore smooth, orange [6B(7–8)] to reddish orange [7B(7–8)], turning reddish black in KOH, rarely cracked; margin thinning out, determinate, adnate, concolorous with or darker than hymenophore surface. Context cream.

Microscopic structures—Hyphal system monomitic; generative hyphae simple-septate. Subiculum distinct; hyphae colorless, slightly to distinctly thick-walled, smooth, usually branched at a right angle, frequently septate, loosely interwoven, 3–6 μm in diam. Subhymenium thickening, composed of collapsed hymenia; hyphae colorless, thin- to slightly thick-walled, vertically arranged, frequently branched and septate, 2–4 μm in diam. Septocystidia abundant, cylindrical, usually sinuous, frequently septate, colorless, slightly thick-walled, smooth, or sometimes slightly encrusted with yellowish granules at the apex, arising from the subiculum, mostly embedded, projecting up to 40 μm beyond hymenium, 145–190 × 6–10 μm. Basidia clavate to subcylindrical, colorless, thin-walled, smooth, with a basal simple septum and four sterigmata, 16–24 × 3.5–5 μm. Basidiospores short-cylindrical to allantoid, with an apiculus, colorless, thin-walled, smooth, IKI–, CB–, 4–5 × 1.5–2 μm, L = 4.5 μm, W = 1.7 μm, Q = 2.6 (n = 30/1).

**Figure 2:**
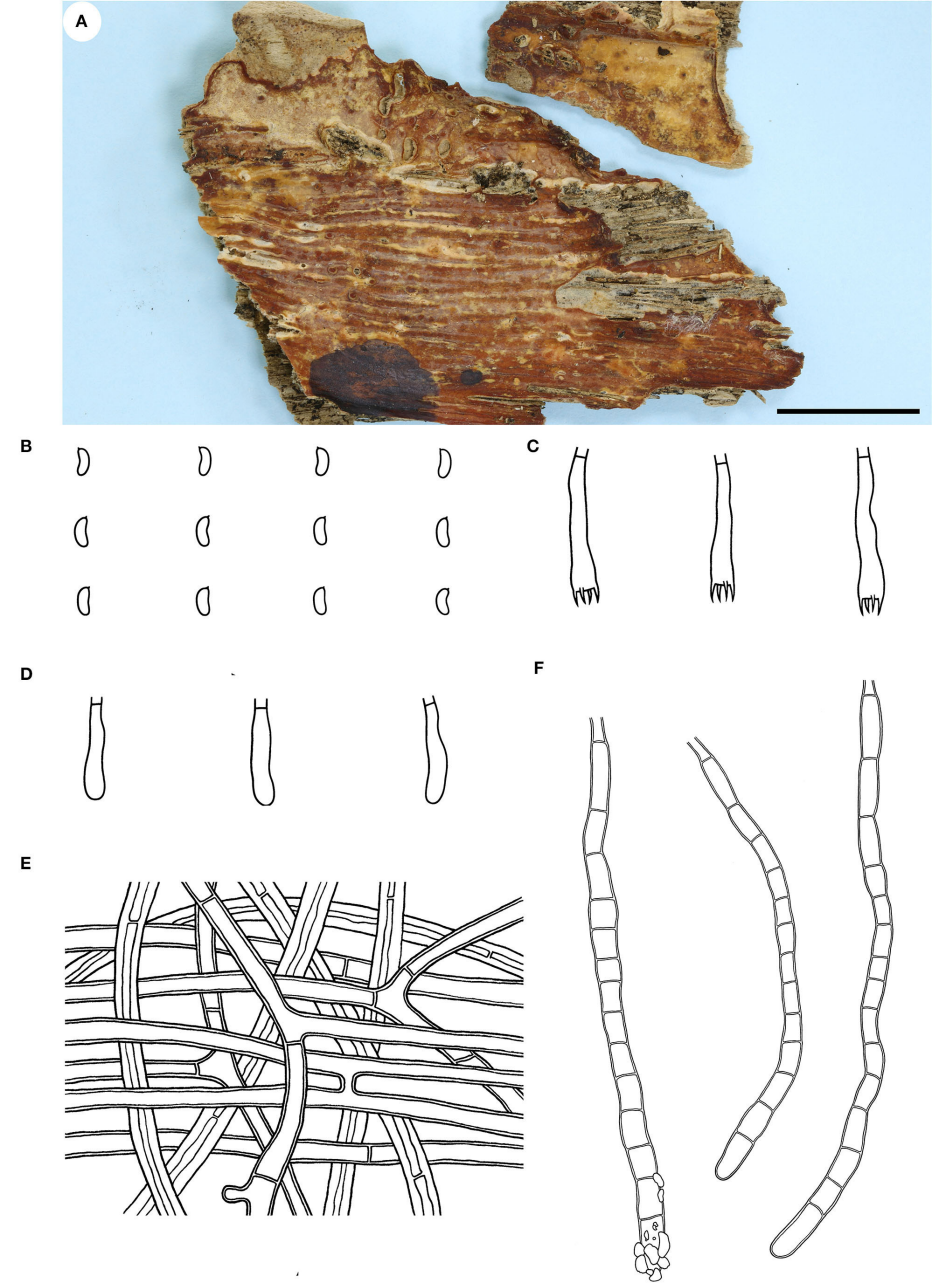
*Candelabrochaete guangdongensis* (from the holotype He 5902; scale bars: a = 1 cm; b–d, f = 10 μm; e = 20 μm). **(A)** Basidiomata. **(B)** Basidiospores. **(C)** Basidia. **(D)** Basidioles. **(E)** Septocystidia. **(F)** Hyphae from subiculum.

Notes—*Candelabrochaete guangdongensis* (Figure 2) is characterized by the ceraceous basidiomata with a smooth hymenophore that turns black in KOH, large septocystidia and short-cylindrical to allantoid basidiospores. In the phylogenetic tree (Figure 1), *C. guangdongensis* is closely related to *C. septocystidia* (Burt) Burds., and morphologically both species have smooth hymenophore, large septocystidia, and allantoid basidiospores. However, *C. septocystidia* can be distinguished from *C. guangdongensis* by having a hymenophore unchanged in KOH, slightly longer basidiospores (4.5–6.5 μm), distinctly encrusted septocystidia, and a distribution in North America and Europe (Burdsall, 1984). *Candelabrochaete macaronesica* M. Dueñas, Tellería and Melo is similar to *C. guangdongensis* by sharing smooth hymenophore and large septocystidia but differs in having slightly larger ellipsoid basidiospores and a distribution in Portugal (5–6.5 × 2–3 μm, Dueñas et al., 2008). *Candelabrochaete neocaledonica* Duhem and Buyck has similar septocystidia and basidiospores with *C. guangdongensis* but differs in having a distinctly hydnoid hymenophore (Duhem and Buyck, 2011).

#### *Efibula grandinosa* Y. Li and S.H. He, sp. nov.

MycoBank: MB843532

Type—China, Yunnan Province, Shizong County, Junzishan Forest Park, on dead angiosperm branch, November 18, 2019, He 6312 (BJFC 033256, holotype).

Etymology—Refers to the grandinioid hymenophore due to the projecting hyphal pegs.

Fruiting body—Basidiomata annual, resupinate, widely effused, closely adnate, inseparable from substrate, membranaceous, first as small patches, later confluent up to 10-cm long, 3-cm wide, up to 250-μm thick in section. Hymenophore grandinioid with projecting hyphal pegs, pale orange (6A3) to grayish orange [6B(4–5)], slightly darkening in KOH, not cracking upon drying; margin thinning out, adnate, indistinct, paler than hymenophore surface, white. Context orange white.

Microscopic structures—Hyphal system monomitic; generative hyphae simple-septate. Subiculum distinct; hyphae colorless, thin- to slightly thick-walled, smooth, moderately branched, frequently septate, loosely interwoven, 2.5–3.8 μm in diam. Subhymenium thin; hyphae colorless, thin-walled, smooth, moderately branched and septate, interwoven. Cystidia absent. Hyphal pegs scattered, largely projecting beyond hymenium, composed of many vertically arranged hyphae; hyphae colorless, thin-walled, smooth or occasionally encrusted with crystals, unbranched, infrequently septate. Basidia clavate, colorless, thin-walled, smooth, with a basal simple septum and four sterigmata, 36–43 × 5–7 μm. Basidiospores ellipsoid, with an apiculus, colorless, thin-walled, smooth, IKI–, CB–, 6–6.8 (−7) × (3.5–) 3.7–4 (−4.1) μm, L = 6.4 μm, W = 3.9 μm, Q = 1.7 (*n* = 30/1).

**Figure 3:**
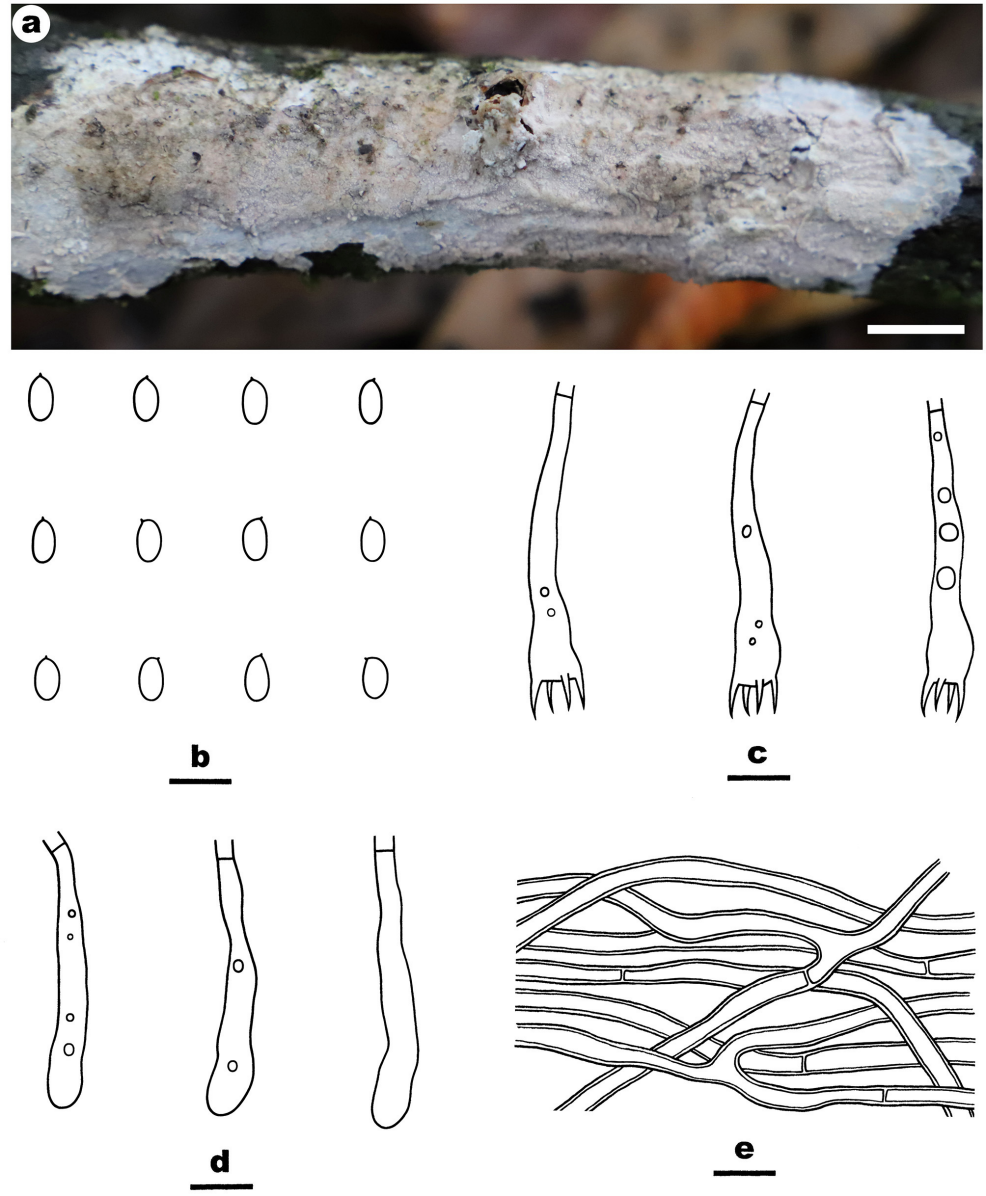
*Efibula grandinosa* (from the holotype He 6312; scale bars: a = 1 cm; b–e = 10 μm). **(A)** Basidiomata. **(B)** Basidiospores. **(C)** Basidia. **(D)** Basidioles. **(E)** Hyphae from subiculum.

Notes—*Efibula grandinosa* Figure 3 is characterized by having a grandinioid hymenophore and hyphal pegs. In the phylogenetic tree (Figure 1), *E. grandinosa* and *E. shenghuae* formed a strongly supported lineage sister to *E. clarkii* Floudas and Hibbett. Morphologically, although all the three species have grandinioid or tuberculate hymenophore, *E. shenghuae* and *E. clarkii* can be easily distinguished from *E. grandinosa* by lacking hyphal pegs (Floudas and Hibbett, 2015).

#### *Efibula hainanensis* Y. Li and S.H. He, sp. nov.

MycoBank: MB843533

Type—China, Hainan Province, Changjiang County, Bawangling Nature Reserve, on dead liana, July 4, 2019, He 6004 (BJFC 030880, holotype).

Etymology—Refers to the type locality in Hainan Province, southern China.

Fruiting body—Basidiomata annual, resupinate, widely effused, closely adnate, inseparable from substrate, membranaceous, first as small patches, later confluent up to 8-cm long, 2.5-cm wide, up to 200-μm thick in section. Hymenophore smooth, pale orange (5A3) to brownish orange [5C(4–5)], turning black in KOH, not cracking upon drying; margin thinning out, adnate, indistinct, paler than hymenophore surface, white to pale yellow. Context pale yellow.

Microscopic structures—Hyphal system monomitic; generative hyphae simple-septate. Subiculum distinct; hyphae colorless, thin- to thick-walled, usually encrusted with small crystals, straight, rarely branched, infrequently septate, loosely interwoven, 1.8–3.5 μm in diam. Subhymenium thin; hyphae colorless, thin-walled, encrusted with fine crystals, moderately septate. Cystidia rare, subfusiform to subcylindrical, colorless, thin-walled, smooth, with a basal simple septum, embedded or slightly projecting beyond the hymenium, 30–50 × 5–9 μm. Basidia clavate, colorless, thin-walled, smooth, with a basal simple septum and four sterigmata, 15–26 × 4–6 μm. Basidiospores ellipsoid to broadly ellipsoid, with an apiculus, colorless, thin-walled, smooth, IKI–, CB–, (4–) 4.2–5.5 (−5.8) × (2.5–) 2.8–3.2 (−3.5) μm, L = 4.7 μm, W = 3.0 μm, Q = 1.6 (n = 30/1).

Additional specimen examined—China, Taiwan, Taitung, Orchid Island, Tienchih, on branch of angiosperm, May 16, 2003; Chen 1284 (TNM F0015135).

**Figure 4:**
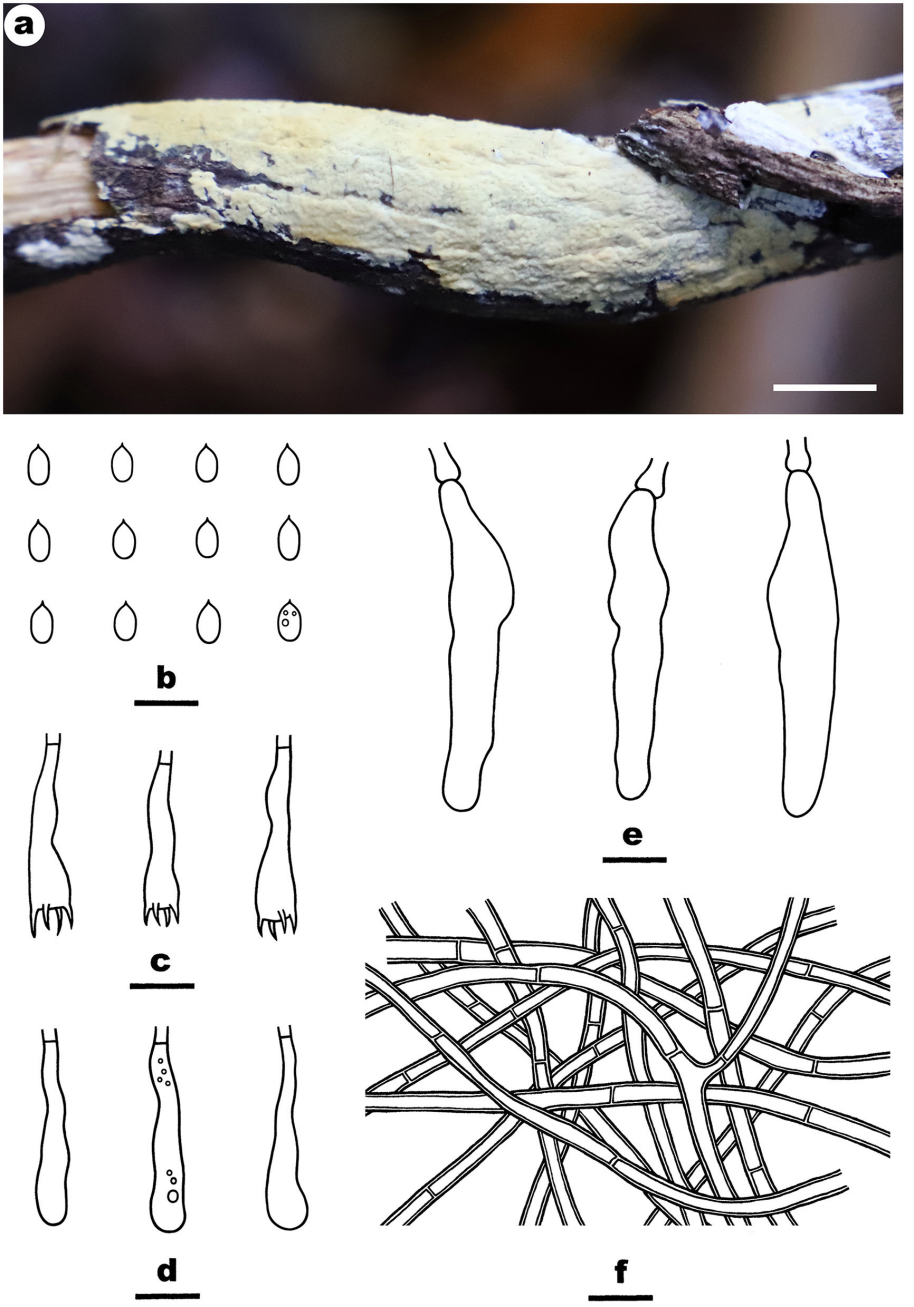
*Efibula hainanensis* (from the holotype He 6004; scale bars: a = 1 cm; b–f = 10 μm). **(A)** Basidiomata. **(B)** Basidiospores. **(C)** Basidia. **(D)** Basidioles. **(E)** Cystidia. **(F)** Hyphae from subiculum.

Notes—*Efibula hainanensis* Figure 4 is characterized by having a smooth hymenophore, thin-walled cystidia, and relatively small ellipsoid basidispores. Until now, *E. hainanensis* is the only species in the genus with cystidia. In the phylogenetic tree (Figure 1), *E. hainanensis* and *E. intertexta* (Sheng H. Wu) C.C. Chen and Sheng H. Wu formed a relatively strongly supported lineage. Morphologically, *E. intertexta* differs from *E. hainanensis* by having distinctly thickening hymenial layer and cylindrical basidiospores (5.6–6.4 × 2.2–2.6 μm) and lacking cystidia (Chen et al., 2021). *Efibula tuberculata* (P. Karst.) Zmitr. and Spirin is similar to *E. hainanensis* by sharing relatively small basidiospores but differs in having a smooth to tuberculate, cracked hymenophore, occasional clamps, and slightly larger basidiospores (Chen et al., 2021).

#### *Efibula shenghuae* Y. Li and S.H. He, sp. nov.

MycoBank: MB843534

Type—China, Yunnan Province, Baoshan County, Gaoligongshan Nature Reserve, on dead branch of *Quercus*, November 30, 2015, He 3384 (BJFC 021779, holotype; isotype in CFMR).

Etymology—Named to honor Dr. Sheng-Hua Wu (TNM, Taiwan) who established and contributed much to the genus of *Efibula*.

Fruiting body—Basidiomata annual, resupinate, widely effused, closely adnate, inseparable from substrate, membranaceous, first as small patches, later confluent up to 8-cm long, 1.5-cm wide, up to 200-μm thick in section. Hymenophore smooth to grandinioid with irregular and scattered granules, orange white (5A2) to pale orange (5A3), unchanged in KOH, not cracking upon drying; margin thinning out, adnate, indistinct, fimbriate, paler than hymenophore, white. Context white.

Microscopic structures—Hyphal system monomitic; generative hyphae simple-septate. Subiculum indistinct, thin; hyphae colorless, slightly thick-walled, smooth, rarely branched, moderately septate, more or less parallel to substrate, 3–4.5 μm in diam. Subhymenium distinct, thickening, with masses of crystals; hyphae colorless, thin-walled, smooth, densely interwoven, agglutinated, frequently septate, 1.8–3.5 μm in diam. Cystidia absent. Basidia clavate, colorless, thin-walled, smooth, with a basal simple septum and four sterigmata, 23–38 × 4.5–7 μm. Basidiospores oblong ellipsoid, with an apiculus, colorless, thin-walled, smooth, IKI–, CB–, 6–6.5 (−6.8) × 3–3.5 (−3.8) μm, L = 6.2 μm, W = 3.2 μm, Q = 1.9 (n = 30/1).

**Figure 5:**
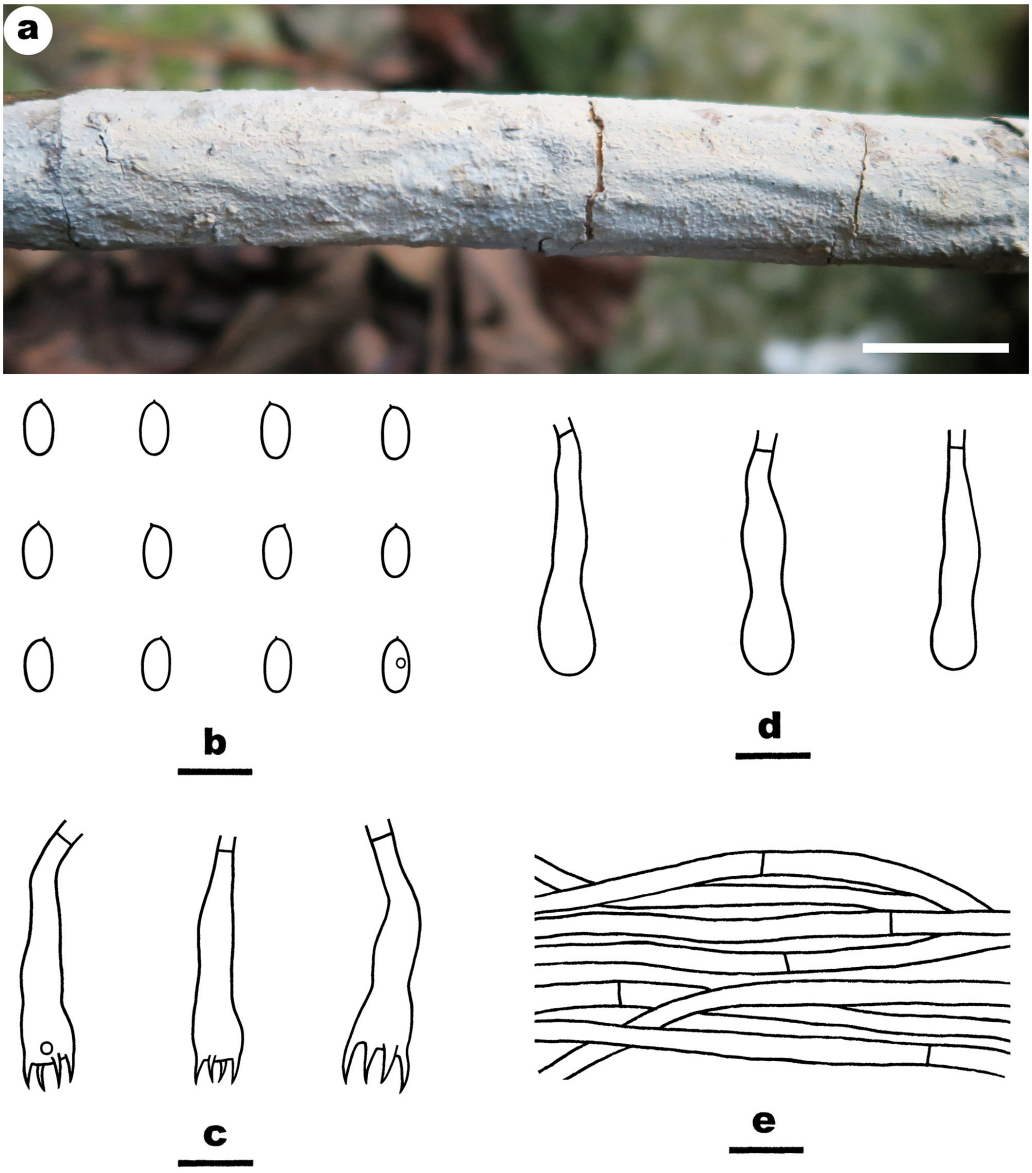
*Efibula shenghuae* (from the holotype He 3384; scale bars: a = 1 cm; b–e = 10 μm). **(A)** Basidiomata. **(B)** Basidiospores. **(C)** Basidia. **(D)** Basidioles. **(E)** Hyphae from subiculum.

Notes—*Efibula shenghuae* Figure 5 is characterized by its grandinioid hymenophore, indistinct subiculum, and masses of crystals in subhymenium. In the phylogenetic tree (Figure 1), *E. shenghuae* and *E. grandinosa* formed a well-supported lineage sister to *E. clarkii*. However, *E. grandinosa* can be easily distinguished from *E. shenghuae* by having hyphal pegs, while *E. clarkii* differs from *E. shenghuae* by having extensively cracked basidiomata and less crystals (Floudas and Hibbett, 2015). *Efibula tuberculata* is similar to *E. shenghuae* by sharing smooth to tuberculate hymenophore but differs by having cracked basidiomata and less crystals in section (Chen et al., 2021). Moreover, the two species formed distinct lineages in the tree.

#### *Efibula taiwanensis* Y. Li and S.H. He, sp. nov.

MycoBank: MB843535

Type—China, Taiwan, Nantou County, Lianhuachi Forest Park, on dead angiosperm branch, December 6, 2016, He 4582a (BJFC 024024, holotype).

Etymology—Refers to the type locality in Taiwan.

Fruiting body—Basidiomata annual, resupinate, widely effused, closely adnate, inseparable from substrate, membranaceous to slightly pellicular, first as small patches, later confluent up to 6-cm long, 1.5-cm wide, up to 150-μm thick in section. Hymenophore smooth, white (5A1) to orange white (5A2), unchanged in KOH, not cracking upon drying; margin thinning out, adnate, indistinct, arachnoid, paler than hymenophore surface, white. Context white.

Microscopic structures—Hyphal system monomitic; generative hyphae simple-septate. Subiculum distinct, thick; hyphae colorless, thin- to slightly thick-walled, smooth or slightly to heavily encrusted with fine crystals, loosely interwoven, moderately branched and septate, 2.5–4 μm in diam. Subhymenium thin; hyphae colorless, thin-walled, smooth or slightly encrusted with fine crystals, interwoven, 2–3.5 μm in diam. Cystidia absent. Basidia clavate to subcylindrical, colorless, thin-walled, smooth, with a basal simple septum and four sterigmata, 24–44 × 6–8 μm. Basidiospores broadly ellipsoid to ovoid, with a distinct apiculus, colorless, thin-walled, smooth, IKI–, CB–, (5.5–) 5.8–6.5 (−7) × 4–4.5 μm, L = 6.2 μm, W = 4.2 μm, Q = 1.5 (n = 30/1).

**Figure 6:**
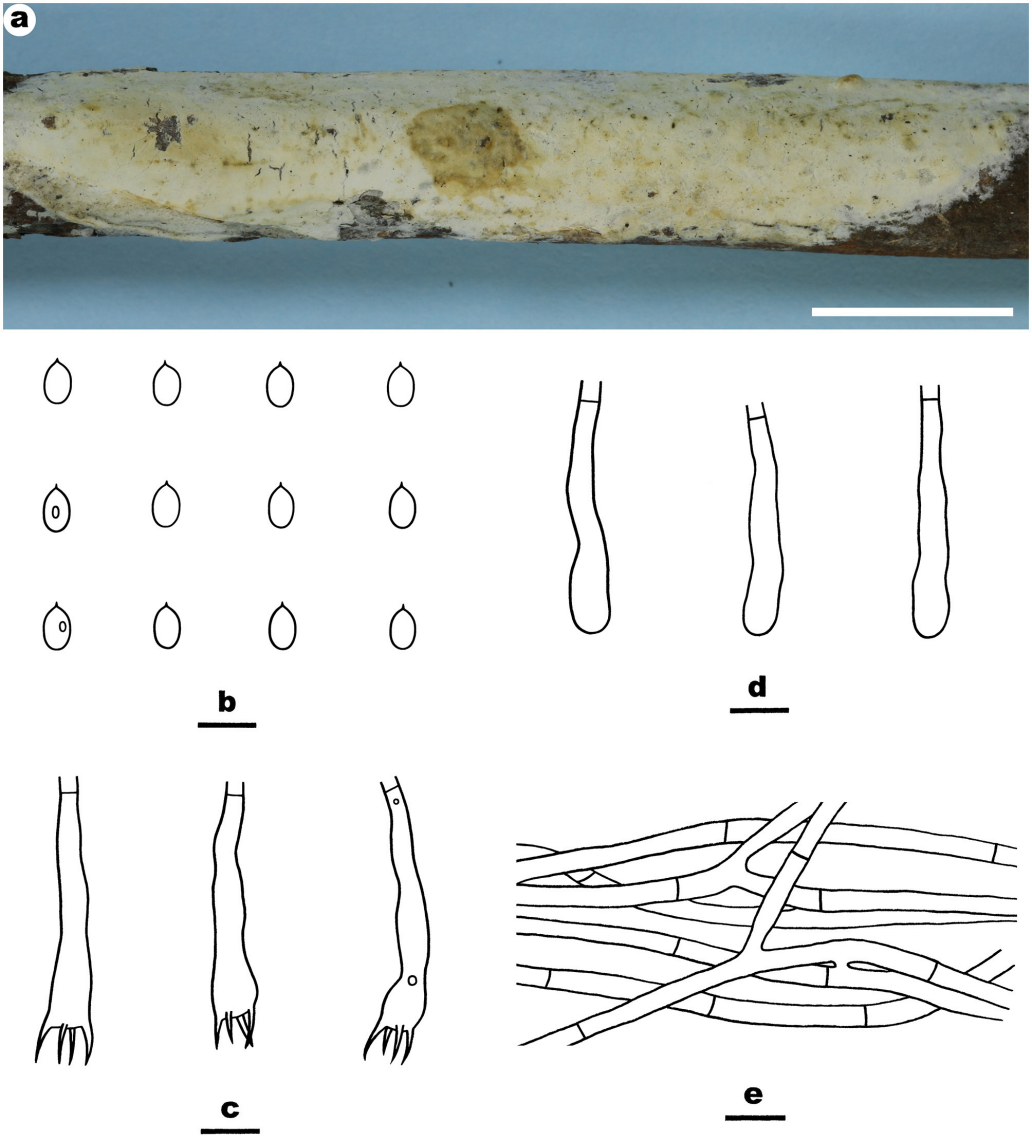
*Efibula taiwanensis* (from the holotype He 4582a; scale bars: a = 1 cm; b–e = 10 μm). **(A)** Basidiomata. **(B)** Basidiospores. **(C)** Basidia. **(D)** Basidioles. **(E)** Hyphae from subiculum.

Notes—*Efibula taiwanensis* Figure 6 is characterized by having membranaceous to pellicular basidiomata and broadly ellipsoid to ovoid basidiospores. In the phylogenetic tree, *E. taiwanensis* is sister to *E. subglobispora* C.C. Chen and Sheng H. Wu and *E. americana* Floudas and Hibbett. Morphologically, *E. subglobispora* differs from *E. taiwanensis* by having larger basidiospores (6.4–8.1 × 4.5–5.8 μm, Chen et al., 2021), while *E. americana* can be easily distinguished from *E. taiwanensis* by having smaller ellipsoid to cylindrical basidiospores (5.3–6.5 × 3–3.8) and a distribution in USA (Floudas and Hibbett, 2015).

#### *Irpex alboflavescens* Y. Li, Nakasone and S.H. He, sp. nov.

MycoBank: MB843536

Type—China, Hainan Province, Wuzhishan County, Wuzhishan Nature Reserve, on fallen angiosperm trunk, June 10, 2016, He 3933 (BJFC 022435, holotype).

Etymology—Refers to the color of hymenophore, “*albo* (Lat.)” = white; “*flavescens* (Lat.)” = yellow.

Fruiting body—Basidiomata annual, resupinate, widely effused, adnate, separable from substrate, coriaceous, first as small patches, becoming confluent up to 15-cm long, 5-cm wide, up to 350-μm thick in section. Hymenophore smooth or slightly tuberculate when fresh, pale orange (5A3) to grayish orange [5B(4–5)], slightly darkening in KOH, rarely cracked; margin thinning out, adnate or sometimes elevated and curved inside exposing substrate upon drying, indistinct, fimbriate, paler than hymenophore, white to orange white. Context white.

Microscopic structures—Hyphal system monomitic; generative hyphae simple-septate. Subiculum distinct; hyphae colorless, slightly thick-walled, usually encrusted with fine crystals, loosely interwoven, infrequently branched, moderately septate, 2–5 μm in diam. Subhymenium distinct, thickening; composed of lamprocystidia and hyphae; hyphae colorless, slightly thick-walled, smooth, interwoven, slightly agglutinated, moderately branched and septate, 2–4 μm in diam. Lamprocystidia numerous, metuloid, colorless, thick-walled, heavily encrusted, embedded or slightly projecting beyond the hymenium, 35–70 × 8–16 μm (crystals included). Basidia subcylindrical, colorless, thin-walled, smooth, with a basal simple septum and four sterigmata, 13–26 × 3.2–6.5 μm. Basidiospores ellipsoid to broadly ellipsoid, with a distinct apiculus, colorless, thin-walled, smooth, IKI–, CB–, (3.8–) 4.2–5.8 (−6) × (2.5–) 2.8–3.5 (−3.8) μm, L = 4.8 μm, W = 3.1 μm, Q = 1.4–1.6 (*n* = 120/4).

Additional specimens examined—China, Guangxi Autonomous region, Longzhou County, Nonggang Nature Reserve, on dead angiosperm branch, 22 July 2012, He 20120722–4 (BJFC 014505, CFMR); on fallen wood of rattan, June 4, 2015, Dai 15296 (BJFC 019407); Huanjiang County, Mulun Nature Reserve, on dead angiosperm branch, July 10, 2017, He 4719 (BJFC 024238) and He 4724 (BJFC 024243); Guizhou Province, Libo County, Maolan Nature Reserve, on dead angiosperm branch, June 15, 2016, He 3782 (BJFC 022281) and He 3791 (BJFC 022290); Hunan Province, Shimen County, Hupingshan Nature Reserve, on fallen angiosperm trunk, July 6, 2015, He 2278 (BJFC 020733, CFMR); Jiangxi Province, Lianping County, Jiulianshan Nature Reserve, on dead angiosperm branch, August 13, 2016, He 4311 (BJFC 023753). Malaysia, Sembilan, Semenyih, Broga Hill, on dead angiosperm branch, 3 December 2019, He 6355 (BJFC 033299). Sri Lanka, Central Province, Kandy, Udawattakele Royal Forest Park, on dead angiosperm branch, March 2, 2019, He 5767 (BJFC 030634); Avissawella, Salgala Forest, on fallen angiosperm branch, March 3, 2019, He 5783 (BJFC 030650); Western Province, Ingiriya, Dombagaskanda Forest Reserve, on dead angiosperm branch, February 27, 2019, He 5732 (BJFC 030599); Mitirigala Nissarana vanaya Forest Monastery, on dead angiosperm branch, March 4, 2019, He 5835 (BJFC 030702). Thailand, Chiang Rai, Campus of Mae Fah Luang University, on dead angiosperm branch, July 21, 2016, He 4037 (BJFC 023476).

**Figure 7:**
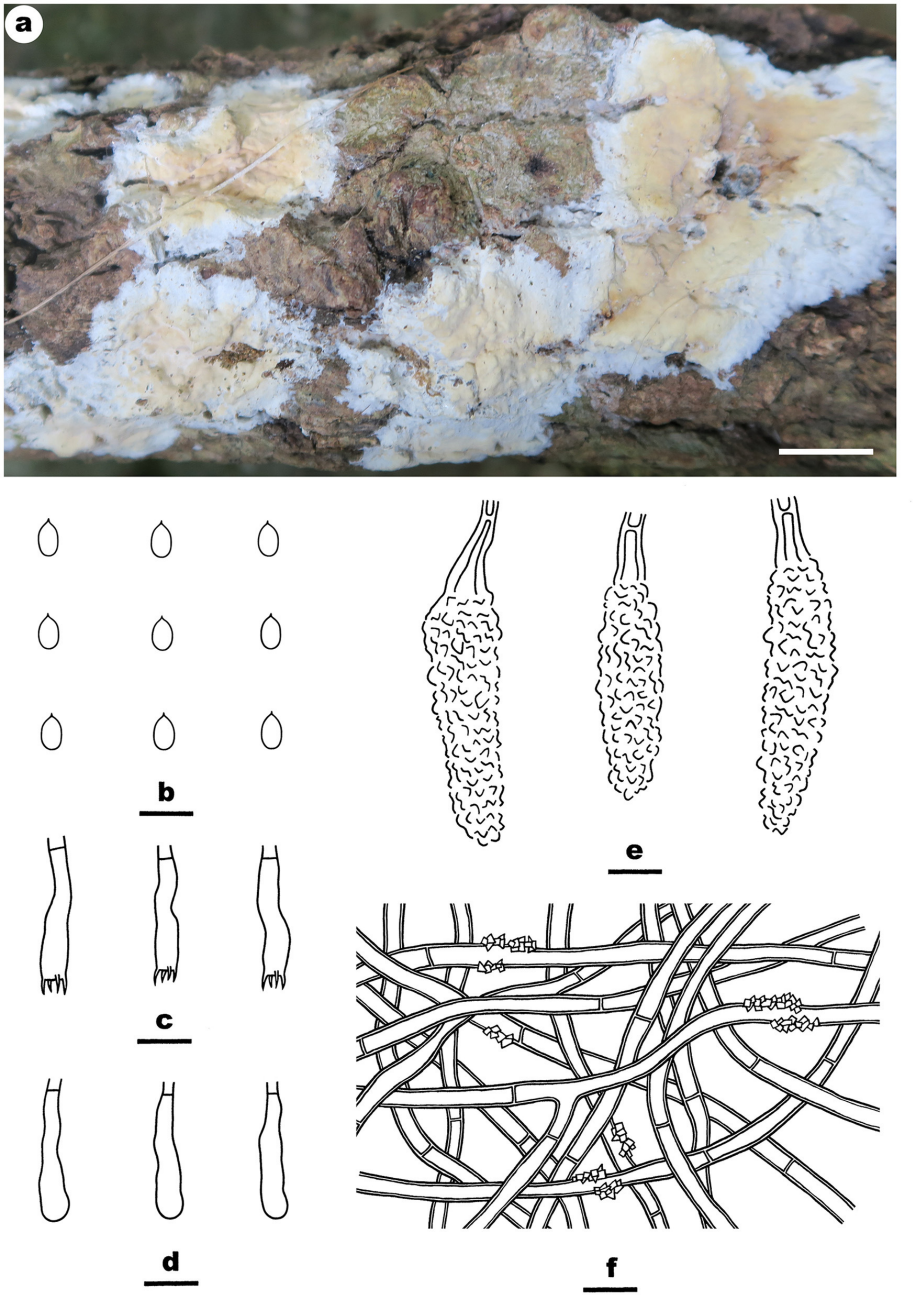
*Irpex alboflavescens* (from the holotype He 3933; scale bars: a = 1 cm; b–f = 10 μm). **(A)** Basidiomata. **(B)** Basidiospores. **(C)** Basidia. **(D)** Basidioles. **(E)** Lamprocystidia. **(F)** Hyphae from subiculum.

**Figure 8:**
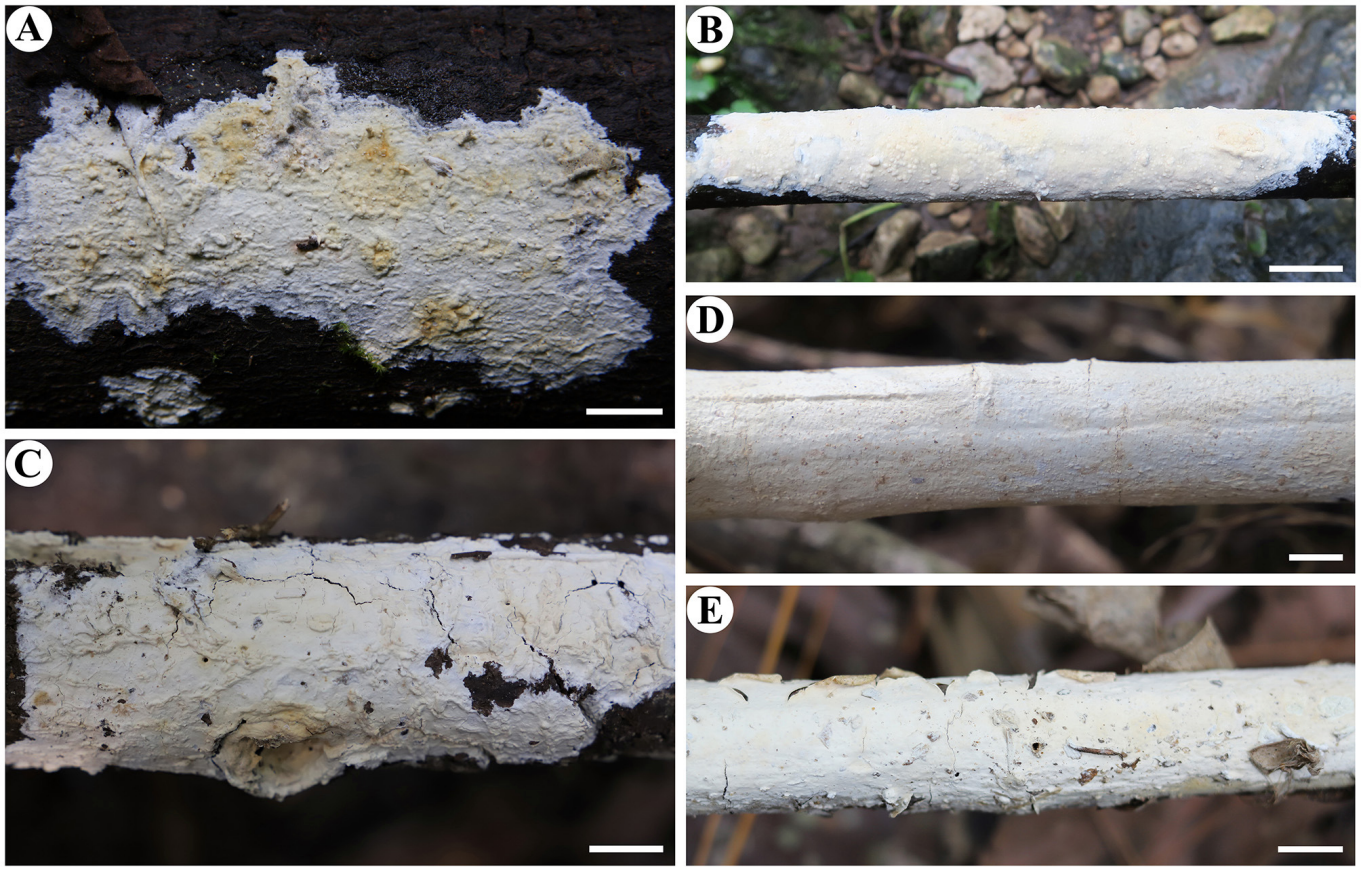
Basidiomata of *Irpex alboflavescens* (scale bars: A–E = 1 cm). **(A)** He 2278; **(B)** He 4719; **(C)** He 5783; **(D)** He 6355; **(E)** He 5732.

Notes—*Irpex alboflavescens* Figures 7, 8 is characterized by having smooth hymenophores, metuloid cystidia, and ellipsoid to broadly ellipsoid basidiospores. In the phylogenetic tree (Figure 1), samples of *I. alboflavescens* from China (including Taiwan), Malaysia, Sri Lanka, Thailand, and USA formed a well-supported distinct lineage sister to *I. lacteus*, the type of the genus. According to the phylogenetic analysis results, Chen et al. (2021) treated *Emmia, Flavodon*, and *Hydnopolyporus* as synonyms of *Irpex*, which now includes species with smooth, poroid, labyrinthine, irpicoid, hydnoid to irregular hymenophore configuration. *Irpex alboflavescens* is similar to *I. lenis* C.C. Chen and Sheng H. Wu that also has a smooth hymenophore, but the latter species differs in lacking cystida and having larger basidiospores (6.2–7.2 × 4.6–5.2 μm, Chen et al., 2021). Moreover, *I. lenis* is phylogenetically distant from *I. alboflavescens* (Figure 1).

#### *Irpex rosea* (C.L. Zhao) Y. Li and S.H. He, comb. nov.

MycoBank: MB844001

= *Flavodontia rosea* C.L. Zhao, Mycotaxon 136: 762, 2022 [2021]. [MB# 838323]

Fruiting body—Basidiomata annual, resupinate to effused–reflexed with slightly elevated margin, adnate, easily separated from substrate, coriaceous, first as small patches, later confluent up to 10-cm long, 3-cm wide, up to 700-μm thick in section. Hymenophore smooth, odontioid or irpicoid, grayish orange [6B(4–5)] to grayish red [7B(4–5)], turning light brown in KOH, rarely cracked; margin thinning out, adnate or slightly elevated and curved inside, indistinct, concolorous with or paler than hymenophore surface. Context cream.

Microscopic structures—Hyphal system monomitic; generative hyphae simple-septate. Basal layer distinct, brown; hyphae yellowish-brown, thick-walled, smooth, agglutinated, parallel to substrate, rarely branched, infrequently septate, up to 7 μm in diam. Subiculum distinct, thick; hyphae colorless, thick-walled, smooth, rarely branched, infrequently septate, slightly agglutinated, parallel to substrate, 3–7 μm in diam. Subhymenium distinct, thick; hyphae colorless, thin-walled, smooth, moderately branched and septate, slightly agglutinated, more or less vertically arranged, 2–4.5 μm in diam. Cystidia absent. Basidia clavate to subcylindrical, colorless, thin-walled, smooth, with a basal simple septum and four sterigmata, 19–28 × 4–6 μm Basidiospores broadly ellipsoid to ovoid, with a distinct apiculus, colorless, thin-walled, smooth, IKI–, CB–, (3.5–) 4–5 (−5.5) × (2.8–) 3–4 μm, L = 4.7 μm, W = 3.4 μm, Q = 1.4 (n = 30/1).

**Figure 9:**
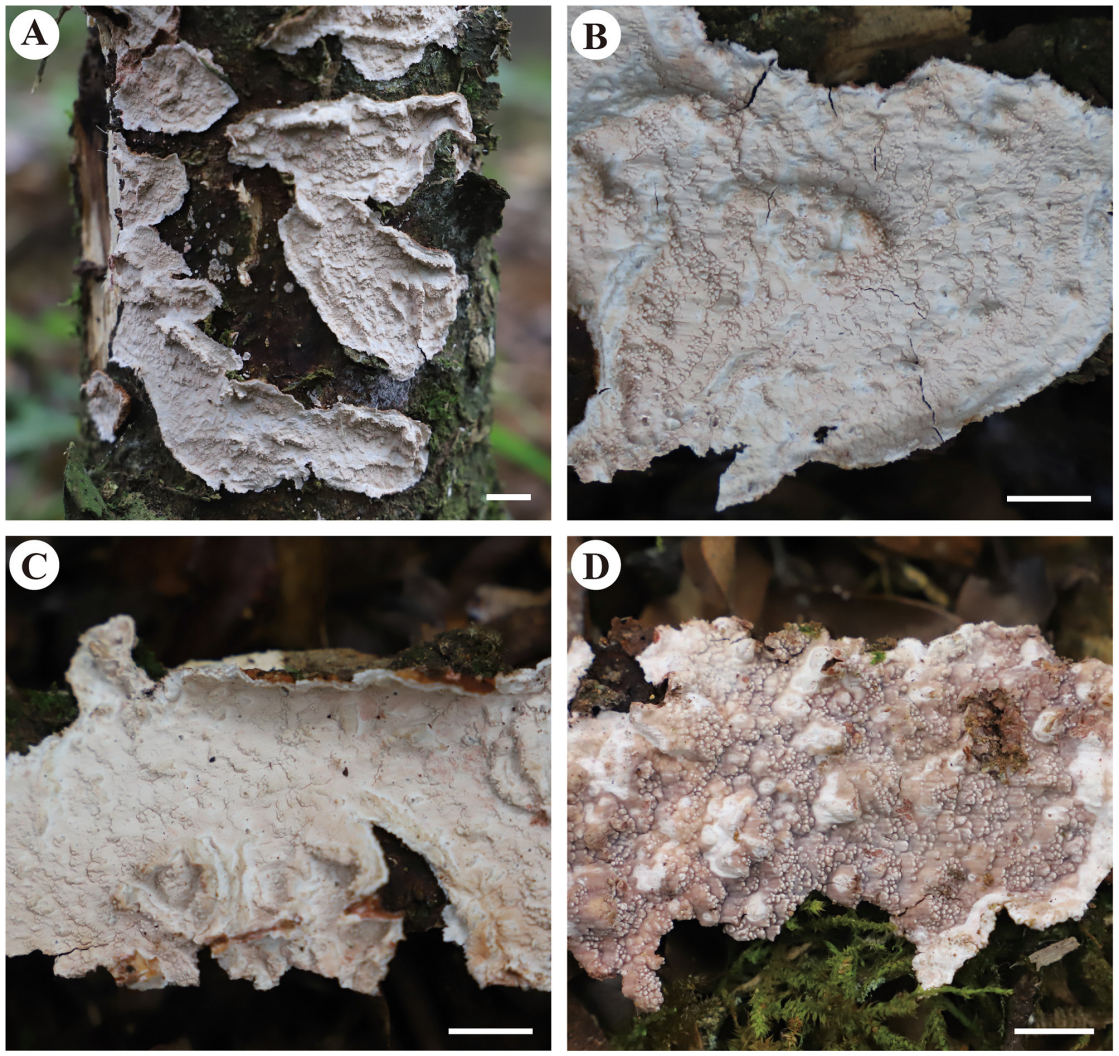
Basidiomata of *Irpex rosea* [from He 6277; scale bars: **(A–D)** = 1 cm].

**Figure 10:**
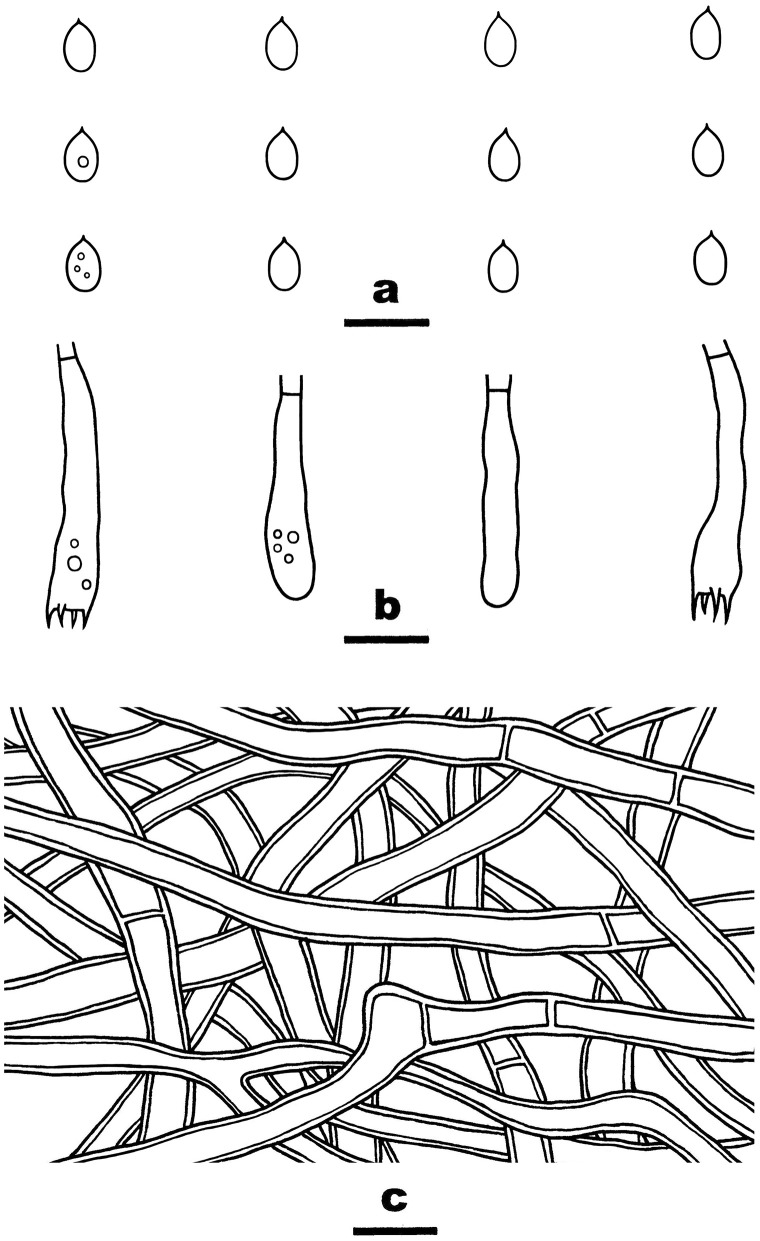
*Irpex rosea* (from He 6277; scale bars: a–c = 10 μm). **(A)** Basidiospores. **(B)** Basidia and basidioles. **(C)** Hyphae from subiculum.

Notes—Wang and Zhao (2022) built a new monotypic genus *Flavodontia* Figures 9, 10 C.L. Zhao for the species *F. rosea* collected from Yunnan Province, southwestern China, mainly based on molecular evidence; however, our phylogenetic analyses using an expanded dataset of Irpicaceae demonstrated that the species was nested within the *Irpex* clade, which has been shown to include taxa from *Emmia, Flavodon*, and *Hydnopolyporus* (Chen et al., 2021). Morphologically, *I. rosea* has effused-reflexed coriaceous basidiomata with smooth, odontioid, or irpicoid hymenophores, simple-septate generative hyphae, broadly ellipsoid basidiospores and lacks cystidia, which fits well with the characters of *Irpex*. Thus, we propose the new combination and treat *Flavodontia* as a later synonym of *Irpex*.

Specimen examined—China, Yunnan Province, Xichou County, Xiaoqiaogou Forest Park, on dead angiosperm stump, November 16, 2019, He 6277 (BJFC 033221).

#### *Phanerochaetella sinensis* Y. Li and S.H. He, sp. nov.

MycoBank: MB843539

Type—China, Jiangxi Province, Yifeng County, Guanshan Nature Reserve, on dead angiosperm branch, August 9, 2016, He 4229 (BJFC 023671, holotype).

Etymology—Refers to the type locality in China.

Fruiting body—Basidiomata annual, resupinate, widely effused, closely adnate, inseparable from substrate, membranaceous to coriaceous, first as small patches, later confluent up to 10-cm long, 2-cm wide, up to 300-μm thick in section. Hymenophore smooth, orange white (5A2) to brownish orange [5C(3–4)], unchanged in KOH, slightly cracked to densely and deeply cracked; margin thinning out or abrupt, adnate or slightly elevated with age, paler than or concolorous with hymenophore. Context white.

Microscopic structures—Hyphal system monomitic; generative hyphae simple-septate. Subiculum indistinct, thin; hyphae colorless, thick-walled, smooth, rarely branched, infrequently septate, more or less agglutinated, parallel to substrate, 3–5 μm in diam. Subhymenium thickening; hyphae colorless, thin- to thick-walled, smooth, moderately branched and septate, agglutinated, densely interwoven, 2.5–4.5 μm in diam. Lamprocystidia arising from subhymenium, narrowly clavate, colorless, thick-walled, heavily encrusted, mostly embedded, 30–70 × 5–10 μm. Basidia clavate to subcylindrical, colorless, thin-walled, smooth, with a basal simple septum and four sterigmata, 20–35 × 4–6 μm. Basidiospores cylindrical, with an apiculus, colorless, thin-walled, smooth, IKI–, CB–, (5.8–) 6–7.2 (−7.8) × 2–3 (−3.2) μm, L = 6.6 μm, W = 2.5 μm, Q = 2.3–2.9 (n = 120/4).

Additional specimens examined—China, Gansu Province, Tianshui County, Maijishan Forest Park, On dead liana, August 8, 2015, He 2484 (BJFC 020937, CFMR); Hubei Province, Wufeng County, Chaibuxi Forest Park, on dead angiosperm branch, August 15, 2017, He 5071 (BJFC 024589) and He 5073 (BJFC 024591); Yunnan Province, Luquan County, Zhuanlong Town, on dead angiosperm branch, December 4, 2015, He 3509 (BJFC 021907, CFMR).

**Figure 11:**
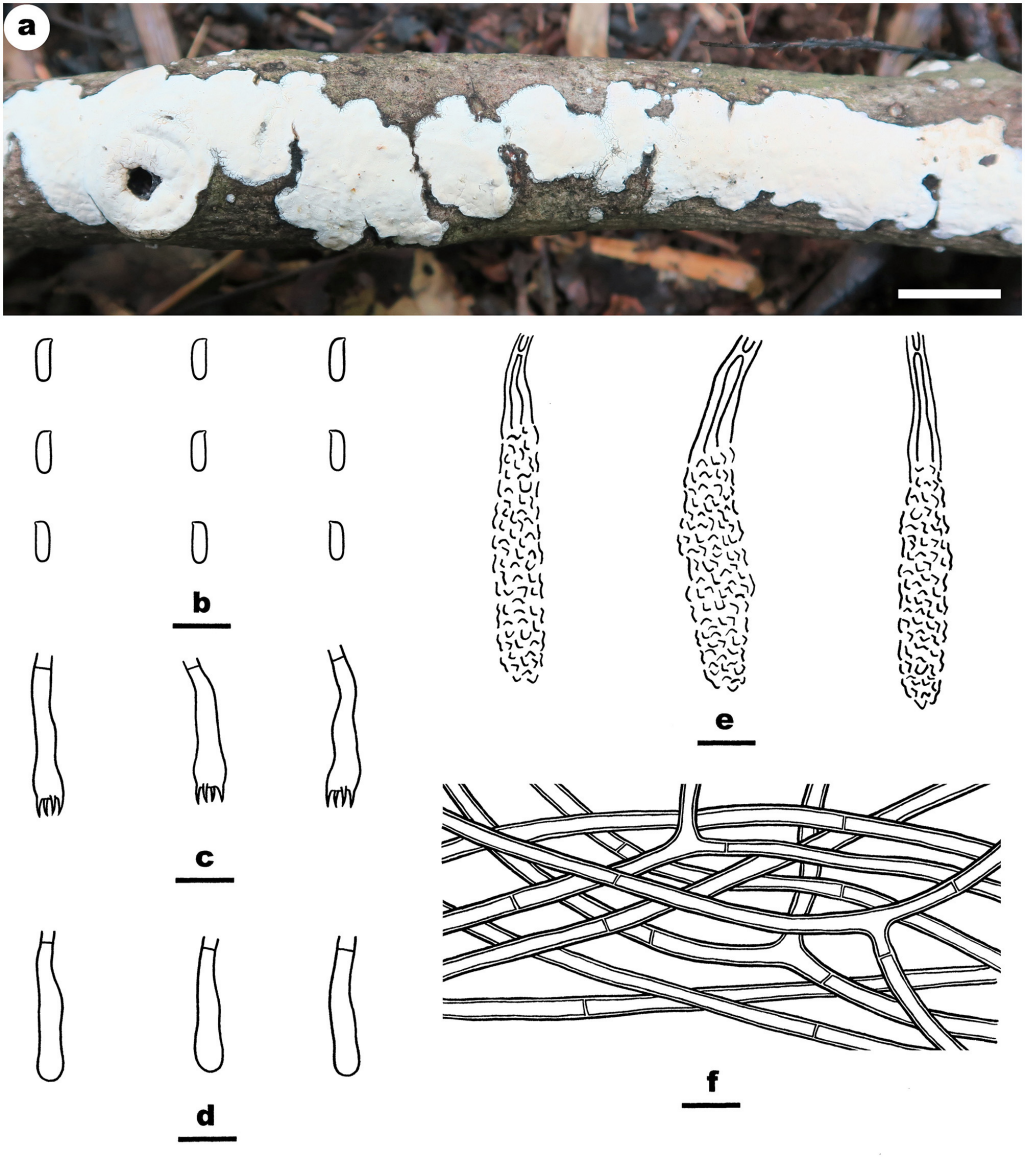
*Phanerochaetella sinensis* (from the holotype He 4229; scale bars: a = 1 cm; b–f = 10 μm). **(A)** Basidiomata. **(B)** Basidiospores. **(C)** Basidia. **(D)** Basidioles. **(E)** Lamprocystidia. **(F)** Hyphae from subiculum.

Notes—*Phanerochaetella sinensis* Figure 11 is characterized having a cracked hymenophore, lamprocystidia, and cylindrical basidiospores. In the phylogenetic tree (Figure 1), three samples of *P. sinensis* formed a distinct lineage sister to *P. exilis* (Burt) C.C. Chen and Sheng H. Wu, which differs in having smaller lamprocystidia (30–50 × 5–6 μm) and ellipsoid basidiospores (5.5–6.5 × 3–3.5 μm, Burdsall, 1985). *Phanerochaetella angustocystidiata* and *P. sinensis* are almost inseparable in micromorphology, but differing in color (ivory or light cream in the former) and thickness (up to 200-μm thick in the former). Meanwhile, they formed distinct lineages in the tree (Wu, 2000).

#### *Phanerochaetella queletii* (Bres.) Y. Li, Nakasone and S.H. He, comb. nov.

MycoBank: MB844002

= *Corticium queletii* Bres., Nuovo Giornale Boanico Italiano 8: 10, 1901. [MB# 183260]

= *Phanerochaete queletii* (Bres.) Nakasone, Cryptogamie Mycologie 29(3): 237, 2008. [MB# 512577]

= *Phanerochaete jose-ferreirae* (D.A. Reid) D.A. Reid, Acta Botanica Croatica 34: 135, 1975. [MB# 319714]

=*Corticium jose-ferreirae* D.A. Ried, Revista Biolgia 5: 140, 1965. [MB# 311853]

Fruiting body—Basidiomata annual, resupinate, widely effused, adnate, separable from substrate, membranaceous to coriaceous, first as small patches, later confluent up to 8-cm long, 4-cm wide, up to 350-μm thick in section. Hymenophore smooth, pale orange (5A3) to grayish orange [5B(4–5)], unchanged in KOH, not cracking or slightly cracked with age; margin thinning out, adnate or loose and slightly elevated with age, fimbriate and paler than hymenophore surface when juvenile, becoming indistinct and concolorous when mature. Context white.

Microscopic structures—Hyphal system monomitic; generative hyphae simple-septate. Subiculum distinct, up to 250-μm thick; hyphae colorless, thin- to slightly thick-walled, slightly encrusted with fine crystals, infrequently branched, moderately septate, loosely interwoven, 2.5–5 μm in diam. Subhymenium indistinct, thin; hyphae colorless, thin-walled, smooth, infrequently branched, moderately septate, loosely interwoven, 2–4.5 μm in diam. Cystidia absent. Basidia clavate to subcylindrical, colorless, thin-walled, smooth, with a basal simple septum and four sterigmata, 28–42 × 4.5–7 μm. Basidiospores cylindrical, with an apiculus, colorless, thin-walled, smooth, IKI–, CB–, (6.8–) 7–8 (−8.2) × 2–2.8 (−3) μm, L = 7.6 μm, W = 2.4 μm, Q = 3.2–3.3 (*n* = 90/3).

Type specimens examined—Italy, Vallombrosa, ad ramos corticates Abietis pectinate, Nov 1899, Martielli (BPI 0282568, holotype of *C. queletii*). Portugal, Serra da Arrabida, on (bark of) fallen branch, 10 May 1964, D.A. Reid (K(M) 146494, holotype of *C. jose-ferrieriae*).

Additional specimens examined—China, Beijing, Mentougou District, Lingshan Scenic Spot, on dead angiosperm branch, April 10, 2022, He 7467 (BJFC 038602); Inner Mongolia Autonomous Region, Genhe County, Greater Khingan Nature Reserve, on dead *Salix* branch, October 17, 2015, He 3050 (BJFC 021440, CFMR); Yakeshi County, Tulihe Forest Park, on dead *Salix* branch, October 18, 2015, He 3065 (BJFC 021455, CFMR); Sichuan Province, Xiaojin County, Jiajin Mountains, on dead angiosperm branch, September 17, 2012, He 20120917-10 (BJFC 014601); Yunnan Province, Baoshan County, Gaoligongshan Nature Reserve, On dead branch of *Alniphyllum*, November 28, 2015, He 3284 (BJFC 021679, CFMR).

**Figure 12:**
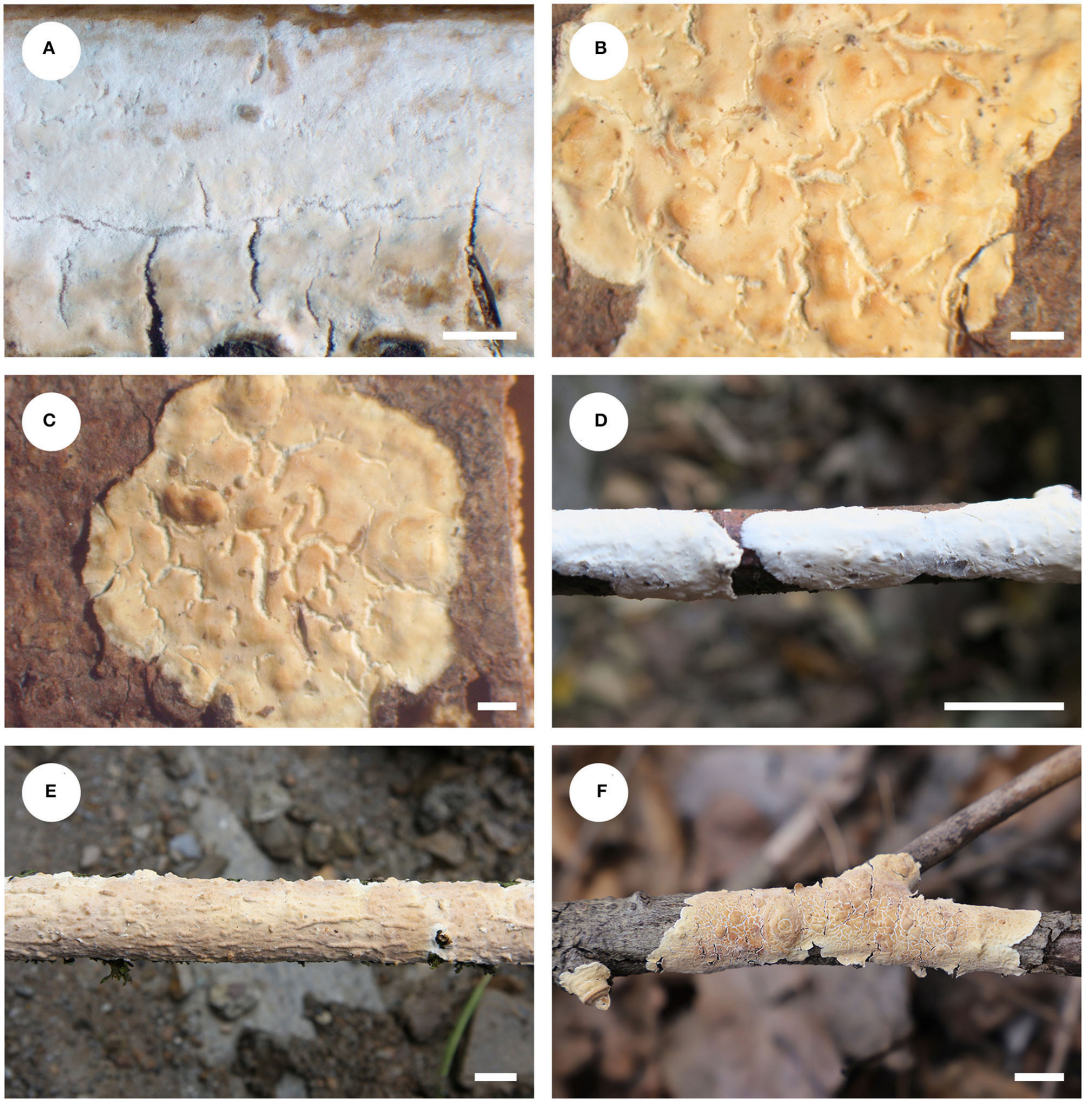
Basidiomata of *Phanerochaetella queletii* (scale bars: A–F = 1 cm). **(A)** K(M) 146494 (holotype of *Corticium jose-ferrieriae*); **(B)** BPI 282568 (holotype of *C. queletii*); **(C)** F11364 (isotype of *C. queletii*); **(D)** He 3284; **(E)** He 20120917-10; **(F)** He 7467.

**Figure 13:**
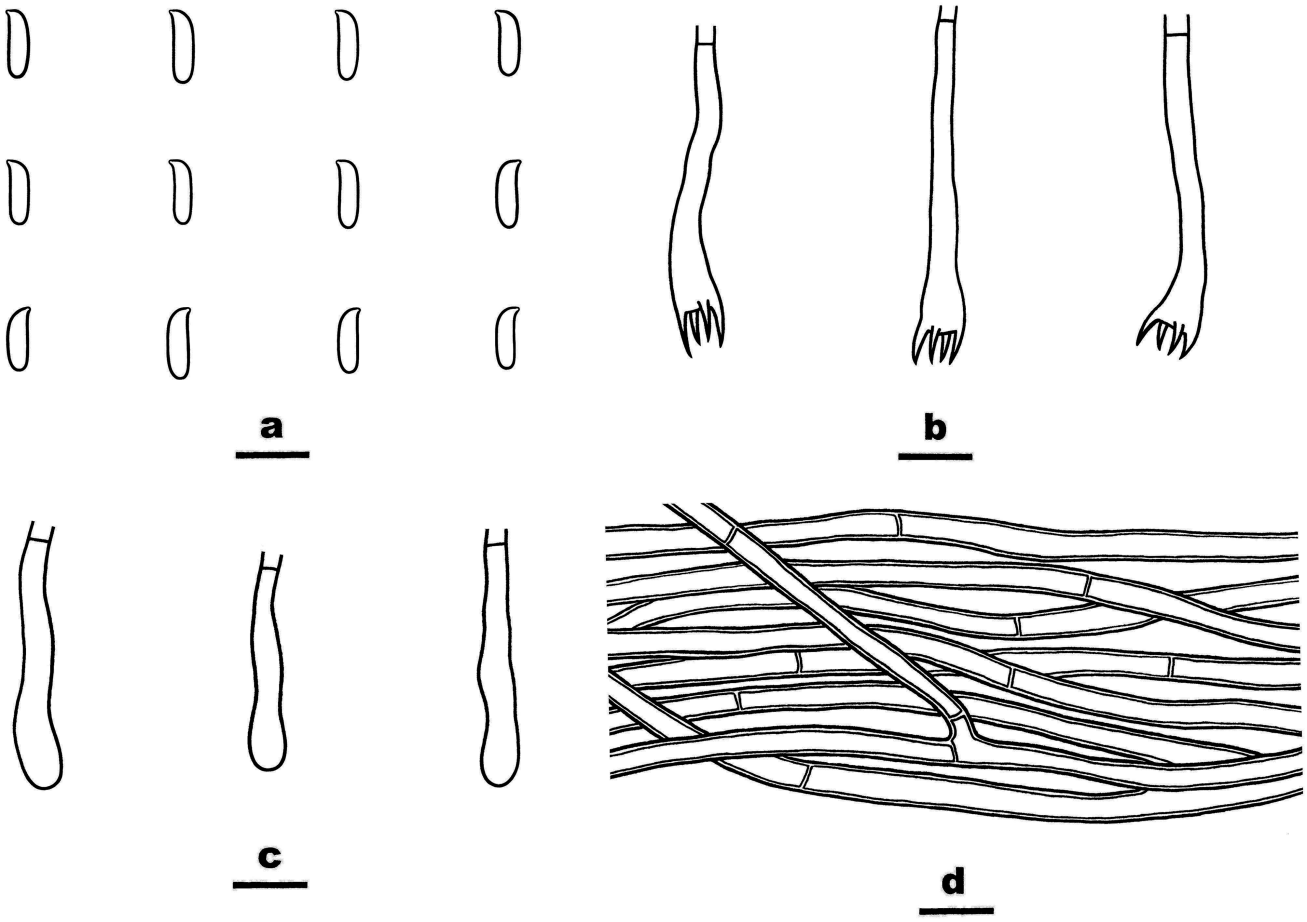
*Phanerochaetella queletii* (from He 3284; scale bars: a–d = 10 μm). **(A)** Basidiospores. **(B)** Basidia. **(C)** Basidioles. **(D)** Hyphae from subiculum.

Notes—*Phanerochaetella queletii* Figures 12, 13 is characterized by basidiomata with smooth to tuberculate hymenophores that often are rimose to reveal the white context and margins that are typically distinct and abrupt that often are slightly detached and incurved, cylindrical basidiospores, and lacking cystidia. Its preferred habitat is small, corticate branches of woody angiosperms, rarely on gymnosperms. This widely distributed species was first described from Italy but is known throughout Europe and is frequently collected in the upper Midwest in the USA. This is the first record this species in China. There is variability in the hymenophore ranging from smooth to distinctly tuberculate, sparsely to highly rimose, and nearly white and cream to brownish orange. Similarly, the margin may be narrowly adnate, white, and fimbriate to abrupt, slightly detached and incurved. Variability in basidiospore length and width was also observed. The description above is based on the Chinese specimens only that appear to have slightly narrower basidiospores than reported earlier. Despite the basidiospore size difference between the type specimens of *C. queletii* and *C. jose-ferrieriae*, their overwhelming similarities in basidiomata texture and color, hymenophore configuration, and other microscopic features indicate that they are conspecific. For additional descriptions and illustrations, see Eriksson et al. (1978), Nakasone (2008), and Bernicchia and Gorjón (2010).

In the phylogenetic tree (Figure 1), three samples of *P. queletii* from China and one sample from USA (HHB-11463) formed a strongly support lineage, which is sister to *P. xerophila* (Burds.) C.C. Chen and Sheng H. Wu from the Sonoran Desert on southwestern USA. and *P. angustocystidiata* (Sheng H. Wu) C.C. Chen and Sheng H. Wu from East Asia. Morphologically, both *P. xerophila* and *P. queletii* lack cystidia, but the former species has broadly ellipsoid basidiospores (6–9 × 3.5–4.5 μm, Burdsall, 1985). *Phanerochaetella angustocystidiata* has cylindrical basidiospores that are slightly smaller than in *P. queletii* and develops lamprocystidia (Wu, 2000).

## Discussion

The species diversity, taxonomy, and phylogeny of the phlebioid clade in Polyporales were intensively studied recently by many authors, and a large number of new taxa from East Asia were described (Floudas and Hibbett, 2015; Miettinen et al., 2016; Justo et al., 2017; Ma and Zhao, 2019; Chen et al., 2020, 2021; Xu et al., 2020; Wang and Zhao, 2021; Zhao et al., 2021; Tian et al., 2022). This study furthers our knowledge of this group with the addition of seven new corticioid species in the Irpicaceae. There is no doubt that more new taxa will be revealed as more surveys are carried out in areas of Asia with the aid of molecular evidence. Our phylogenetic analysis results supported to place the newly described monotypic genus, *Flavodontia*, in synonymy under *Irpex*. We also demonstrated that one species, namely, *P. queletti*, has a wide distribution throughout the north temperate region from China to Europe and North America.

The results of our phylogenetic analyses of Irpicaceae are consistent with that presented by Chen et al. (2021). In both studies, clades of the five genera**—***Irpex, Phanerochaetella, Byssomerulius, Cytidiella*, and *Meruliopsis*, received strong support values, whereas *Efibula* was shown to be paraphyletic with species distributed in four subclades. According to the phylogenetic results, *Irpex* now includes species with smooth, poroid, labyrinthine, irpicoid, hydnoid to irregular hymenophore configurations. However, there are still many old names in *Irpex that* need to be studied by using modern taxonomic methods and systems. For *Efibula*, there are no distinct morphological characters to divide it into small genera at present (Table 2). The newly erected genus, *Phanerochaetella*, contains several species with diverse micromorphology: lamprocystidia present or absent and basidiospores from broadly ellipsoid to cylindrical (Table 3). Three species of *Candelabrochaete* formed two distinct lineages in the *Ceriporia*/*Candelabrochaete* s.l./ *Leptoporus*/*Phanerochaete allantospora* clade, but their generic position remains unresolved since the type species, *C. africana* Boidin, was not nested within the phlebioid clade (Justo et al., 2017; Chen et al., 2021).

**Table 2:** Diagnostic characters for species of *Efibula*.

**Species**	**Hymenophore**	**Color change** **in KOH**	**Cystidia**	**Basidia size** **(μm)**	**Spores**		**Known distribution**	**Reference**
					**Shape**	**Size (μm)**		
*E. americana*	Smooth to reticulate	Not mentioned	Absent	20–32 × 5–8	Ellipsoid to Cylindrical	5.3–6.5 × 3–3.8	USA	Floudas and Hibbett, 2015
*E. clarkii*	Slightly tuberculate	Not mentioned	Absent	25–39 × 5–7.5	oblong to ellipsoid	6–7 × 3–3.5	USA	Floudas and Hibbett, 2015
*E. gracilis*	Smooth	Not mentioned	Absent	17–30 × 5–6.5	Ellipsoid to oblong	5.5–7 × 3.3–4	USA	Floudas and Hibbett, 2015
*E. grandinosa*	grandinioid	Slightly darkening	Absent	36–43 × 5–7	Ellipsoid	6–6.8 × 3.7–4	China	Present study
*E. hainanensis*	Smooth	turning black	rare	15–26 × 4–6	Ellipsoid to broadly ellipsoid	4.2–5.5 × 2.8–3.2	China	Present study
*E. intertexta*	Smooth	No	Absent	30–35 × 4.5–5	Cylindrical	5.6–6.4 × 2.2–2.6	China	Chen et al., 2021
*E. matsuensis*	Smooth	darkening	Absent	18–25 × 6.5–8	Ellipsoid to Cylindrical	7.4–8.6 × 3.8–4.4	China	Chen et al., 2021
*E. rodriguezarmasiae*	Smooth to tuberculate	Not mentioned	Absent	35–48 × 6–8	Ellipsoid	6–7 × 4–5	Micronesia	Boonmee et al., 2021
*E. shenghuae*	Smooth to grandinioid	No	Absent	23–38 × 4.5–7	Oblong ellipsoid	6–6.5 × 3–3.5	China	Present study
*E. subglobispora*	Smooth	No	Absent	30–40 × 6.5–8	Broadly ellipsoid to subglobose	6.4–8.1 × 4.5–5.8	China	Chen et al., 2021
*E. taiwanensis*	Smooth	No	Absent	24–44 × 6–8	Broadly ellipsoid to ovoid	5.8–6.5 × 4–4.5	China	Present study
*E. tropica*	Smooth	No	Absent	20–40 × 5.5–8	Broadly ellipsoid	6.4–7.7 × 3.7–4.4	China and Japan	Chen et al., 2021
*E. tuberculata*	Smooth to slightly tuberculate	No	Absent	18–35 × 5–6	Ellipsoid	5.3–6.4 × 3.4–4.3	East Asia, Europe and North America	Chen et al., 2021
*E. turgida*	Smooth	No	Absent	26–30 × 6.5–7	Cylindrical	6.6–8.2 × 3.3–3.9	China	Chen et al., 2021
*E. yunnanensis*	mainly Smooth, sometimes slightly tuberculate	No	Absent	27–38 × 6–7	Broadly ellipsoid	6.6–8 × 3.9–4.7	China and Japan	Chen et al., 2021

**Table 3:** Diagnostic characters for species of *Phanerochaetella*.

**Species**	**Hymenophore**	**Lamprocystidia (μm)**	**Basidia size (μm)**	**Spores**		**Known distribution**	**Reference**
				**Shape**	**Size (μm)**		
*P. angustocystidiata*	Smooth	Yes; 30–70 × 5–9	18–28 × 4.5–5.5	Cylindrical	6.3–8 × 2.3–3	China and Japan	Wu, 2000
*P. exilis*	Smooth to finely pubescent	Yes; 30–50 × 5–6	12–15 × 5–6	ellipsoid	5.5–6.5 × 3–3.5	Mexico and USA	Burdsall, 1985
*P. formosana*	Smooth	Yes; 20–35 × 7–10	18–24 × 5–6	Cylindrical	7.5–8.7 × 2.9–3.5	China	Chen et al., 2021
*P. leptoderma*	Smooth	Yes; 35–65 × 4–6	21–28 × 4.7–6	Cylindrical	6.4–7.6 × 2.9–3.3	China, India and Japan	Wu, 1990
*P. queletii*	Smooth	No	28–42 × 4.5–7	Cylindrical	7–8 × 2–2.8	China and USA	Present study
*P. sinensis*	Smooth	Yes; 30–70 × 5–10	20–35 × 4–6	Cylindrical	6–7.2 × 2–3	China	Present study
*P. xerophila*	Smooth to tuberculate	No	30–40 × 6–7	Broadly ellipsoid	6–9 × 3.5–4.5	USA	Burdsall, 1985

The molecular evidence has brought significant changes and increased our understanding in the taxonomy of Irpicaceae. The morphological circumscriptions of some genera became broader, for example, *Irpex* now contains species with poroid, labyrinthine, irpicoid, hydnoid to irregular hymenophore, and *Efibula* is shown to contain species with or without horizontally arranged subicular hyphae. Species with simple-septate hyphae and without cystidia can be found in *Efibula, Irpex*, and *Phanerochaetella*. To determine important and useful morphological characters for distinguishing those genera and resolve infra-generic phylogeny, additional taxa from these genera from other regions should be included in the future phylogenetic studies. In addition, comparative morphological analyses of fruitbody features such as subiculum and subhymenium thickness, construction, and texture in addition to basidia, cystidia, and basidiospore shape and size are important areas of consideration in future studies. Information on habitat and distribution may be useful for understanding species delimitation and phylogeny of species within a genus.

## Data Availability Statement

The datasets presented in this study can be found in online repositories. The names of the repository/repositories and accession number(s) can be found below: https://www.ncbi.nlm.nih.gov/genbank/, see the Table 1 included in article.

## Author Contributions

YL performed the phylogenetic analyses and did most of the measurements, descriptions, and illustrations. S-HH designed the research, collected most of the specimens, and wrote the text. C-CC provided with some specimens and sequences. KN examined materials from USA and revised the text. H-XM helped in field trips. All authors contributed to the article and approved the submitted version.

## Funding

Financial support was provided by the National Natural Science Foundation of China (Nos. 31870011 and 31750001).

## Conflict of Interest

The authors declare that the research was conducted in the absence of any commercial or financial relationships that could be construed as a potential conflict of interest.

## Publisher's Note

All claims expressed in this article are solely those of the authors and do not necessarily represent those of their affiliated organizations, or those of the publisher, the editors and the reviewers. Any product that may be evaluated in this article, or claim that may be made by its manufacturer, is not guaranteed or endorsed by the publisher.

## Footnotes

^1^ http://sweetgum.nybg.org/science/ih/

^2^ http://www.biology.duke.edu/fungi/mycolab/primers.htm

^3^ https://www.ncbi.nlm.nih.gov/

^4^ http://mafft.cbrc.jp/alignment/server/

^5^ http://treebase.org/treebase-web/home.html

## Abbreviations

ITS: internal transcribed spacer
nrLSU: nuclear ribosomal large subunit
BJFC, herbarium of Beijing Forestry University, Beijing: China
CFMR, Centre for Forest Mycology Research, U.S. Forest Service, Madison, Wisconsin: USA
TNM, National Museum of Natural Science, Taichung, Taiwan: China
KOH: 2% (w/v) potassium hydroxide
IKI: Melzer's reagent
CB: cotton blue
IKI–: neither amyloid nor dextrinoid
CB–: acyanophilous
L: mean spore length
W: mean spore width
Q, L/W ratio, n (a/b): number of spores (a) measured from number of specimens (b)
CTAB: cetyltrimethylammonium bromide
DNA: deoxyribonucleic acid
PCR: polymerase chain reaction
ML: maximum likelihood
BI: Bayesian inference
BPP: Bayesian posterior probability.
